# On the propagation of nonlinear waves in the atmosphere

**DOI:** 10.1098/rspa.2021.0895

**Published:** 2022-04

**Authors:** A. Constantin, R. S. Johnson

**Affiliations:** ^1^ Faculty of Mathematics, University of Vienna, Oskar-Morgenstern-Platz 1, 1090 Vienna, Austria; ^2^ School of Mathematics, Statistics and Physics, Newcastle University, Newcastle NE1 7RU, UK

**Keywords:** atmospheric waves, asymptotic methods, differential equations

## Abstract

Starting from the general equations of fluid dynamics that describe the atmosphere, and using asymptotic methods, we present the derivation of the leading-order equations for nonlinear wave propagation in the troposphere. The only simplifying assumption is that the flow in the atmosphere exists in a thin shell over a sphere. The systematic approach adopted here enables us to find a consistent balance of terms describing the propagation, and to identify the temperature and pressure gradients that drive the motion, as well as the heat sources required. This produces a new nonlinear propagation equation that is then examined in some detail. With the morning glory in mind, we construct a few exact solutions, which, separately, describe breezes, bores and oscillatory motion.

## 1. Introduction

**Figure 1.**
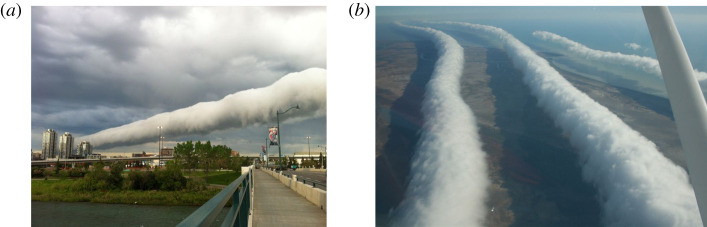
Morning glory cloud patterns composed of a tubular cloud (*a*) or a succession of such roll clouds (*b*). These wave-type patterns travel hundreds of kilometres, propagating horizontally in the direction orthogonal to the cloud line. (*a*) Roll cloud over land, photographed by G. E. Nyland in Calgary on 18 June 2013 (video link: https://m.youtube.com/watch?v=i1rdWjrYG5I). (*b*) Roll cloud photographed over the Gulf of Carpentaria in Northern Australia on 10 August 2009 (source: Mike Petroff, CC BY-SA 3.0). (Online version in colour.)

The ‘morning glory’ is a spectacular cloud pattern composed of a tubular cloud, or succession of such roll clouds, typically stretching from horizon to horizon; see the photographs in figure 1. Comprehensive descriptions of these phenomena using quantitative hypothesis-testing based on field data are available, but a more insightful study requires a careful development from the governing equations of atmospheric flow. Hitherto, the modelling of such local meteorological phenomena has been pursued on a rather ad hoc basis, relying on analogies with shallow-water flow for internal gravity waves in a stably stratified fluid [1–4]. Despite the inadequacies of this approach, important insights have been gained into this phenomenon, in particular indicating that the processes involved are nonlinear [5–7]. We believe, therefore, that a systematic asymptotic derivation of a suitable nonlinear equation that describes the wave propagation is required, starting from the general governing equations. This will provide a reliable test for existing models, which, to be useful, should be consistent with, and can be derived from, the governing equations. More significantly, it will enable us to identify all the relevant factors (such as temperature and pressure gradients, and atmospheric heat sources), and also develop a consistent nonlinear theory.

The plan for this work is as follows. After a brief description of the morning-glory phenomenon (as an important example of nonlinear wave propagation), and the mechanisms that are relevant to its generation, in §2, we then present the general governing equations for the atmosphere in §3. These are the Navier–Stokes equation, the equation of mass conservation, the equation of state for the air and the associated first law of thermodynamics—all written in rotating spherical coordinates. The restriction to spherical geometry is an adequate model for these local phenomena because, in this context, the oblateness of the Earth is of little consequence; see the discussion in [8,9]. However, the use of spherical coordinates is essential if the length scales are anything other than very short—some morning glory waves propagate over 1000 km [10]—and, even more significantly, this enables us to determine higher-order correction terms. Guided by field data for the identification of the relevant scales and parameters, in §4 we perform the non-dimensionalization and scaling of the equations, with the ultimate aim of producing a coherent model for nonlinear wave propagation in the atmosphere. This process involves a rotation of the local coordinates, allowing for propagation in any direction over the Earth, in the thin-shell approximation for the atmosphere. (No other simplifications are used in the development presented here.) The associated asymptotic solution is developed in §5, which involves the perturbation of a background state of the atmosphere, eventually leading to the equation that describes the propagation of a nonlinear wave. This equation is analysed in §6; we are unable to solve it in general, but we make some important observations about it and, in particular, we find a number of exact solutions valid under simplifying assumptions. These show that the new equation captures all the essential elements that describe the evolution of waves such as the morning glory. Indeed, at the stage of the investigation described here, we are able to present solutions, which, although separately and only in certain regions, exhibit features such as the existence of background breezes, bore-like structures and oscillatory motion. It is clear that a comprehensive description of the solutions of this equation will require a study of some complexity, combining mathematical analysis with numerical solutions and simulations; this is far beyond our remit in this initial investigation. Nevertheless, what we present here should provide the basis for future work: local and global well-posedness of the model wave equation is of fundamental importance, as is obtaining further insight into its dynamics. This latter will almost certainly require finding and studying—both qualitatively and quantitatively—other families of solutions.

## 2. The morning glory: observations and genesis mechanism

**Figure 2.**
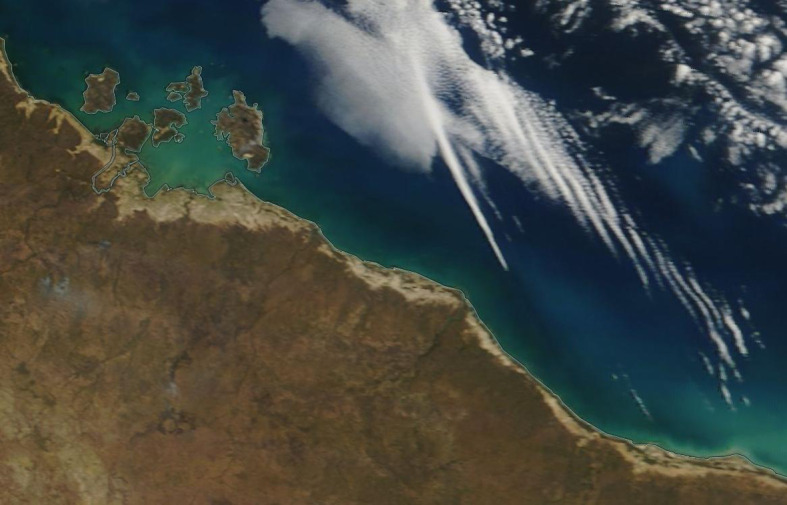
Natural-colour satellite photograph of the ‘morning glory’ cloud formation in the Gulf of Carpentaria (Australia), taken on 18 August 2019 (source: https://worldview.earthdata.nasa.gov). Each roll cloud stretches over more than 50 km, has a width of about 1 km and a cloud base a few 100 m above the ground. The cloud formation propagates in the southwest direction at about $10\;{m\;s}^{-1}$. (Online version in colour.)

Morning glory clouds occur most often in coastal regions but, occasionally, also over the land or the sea (see figures 1, 2 and the photographs in [11–18]). These spectacular formations have been reported as appearing over the English Channel, in Central USA, Germany, Eastern Russia, Canada, Mexico, Brazil, Uruguay and, most particularly, Australia. Here, they can be observed, on a fairly regular basis, in the Gulf of Carpentaria (a large, shallow sea enclosed on three sides, connected northwards to the Arafura Sea between Australia and New Guinea); they are usually observed in the early morning, during September and October. Their appearance in this region is mainly due to the very particular thermal structure of the lower atmosphere over the land and the sea.

**Figure 3.**
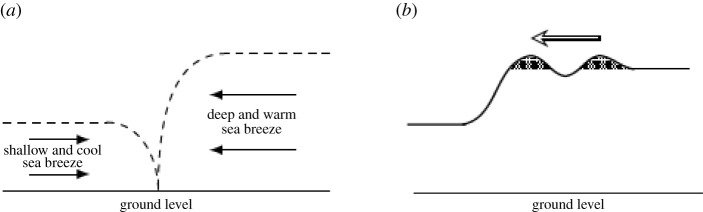
Schematic illustrating the morning-glory cloud-formation-mechanism in the Gulf of Carpentaria. The thick curve in (*b*) represents a snapshot of the top of the thermal inversion layer, normal to the two depicted roll cloud lines, with nearby airflow featuring similar undulations, as confirmed by measurements using pilot-balloon ascents [19]. The cloud lines are not moved by the airflow: they are formed as a wave crest approaches (because ascending air parcels cool and condensation occurs) but, after the wave crest passes, the cloud evaporates (due to the warming of the descending air parcels). The continuous formation and erosion of the clouds gives the appearance that the clouds are rotating [17]: they are referred to as ‘roll clouds’. (*a*) Colliding sea breezes bring about a thermal inversion layer if the warmer breeze reaches a few hundred metres higher than the cooler: a layer of warm air is sandwiched between slightly colder air below and much colder air above it, as the cool breeze pushes the warm air of the other breeze upwards. (*b*) Wave disturbances develop on the top of the thermal inversion layer. The cooling updraft at the front of each wave crest causes condensation of the ascending moist air parcels, with the ensuing cloud (dotted region) eroded by evaporation as the wave crest passes since the descending air parcels warm up.

Firstly, there is a low-lying, wide peninsular—the Cape York peninsular, on the eastern edge of the gulf—which is covered by dry air, sufficiently dry to inhibit the wholesale formation of clouds, but moist enough in the lower layers for cloud lines to form. Secondly, there are two colliding breezes: the offshore breeze forms over the peninsular after sunrise and is driven by the difference in air pressure created by the differing heat capacities of water and dry land; it is warm, moist and slow; the onshore flow is shallower, cooler and faster. The two flows meet just inland at the coast; the offshore wind-speed increases and the flow moves forward and begins to override the onshore breeze: a first wave is formed at the leading edge of the advancing offshore flow. On occasions, only one wave is formed (so we have, essentially, a bore), but fairly often a train of waves appear—perhaps six or seven—each generated at the front and moving in the offshore direction relative to the front; see the sketch in figure 3 and the discussion in [11]. The condensation that occurs within these waves produces the cloud patterns that have been observed. The net effect is to generate local conditions where a warm low-level temperature-inversion layer appears; the temperature in the troposphere, on average, decreases with height, so this inversion layer sits between a slightly colder layer below (in the atmospheric boundary layer) and the much colder atmosphere above; see [19]. Thus, in the lower part of the flow, we have a stably stratified region—warmer air above colder air—in the neighbourhood of the bottom edge of the inversion layer. On the other hand, the unstable stratification near the upper boundary of the inversion layer facilitates horizontal wave propagation, with the lower boundary acting as a waveguide. Eventually, the morning glory clouds vanish as the heat of the day causes them to evaporate.

All the above shows that the morning-glory phenomenon involves a complicated sequence of processes yet, of course, it must be possible to describe the essential features by relying on the governing equations for atmospheric flow. Writing down equations admitting solutions that replicate specific observed flow structures is a relatively routine exercise (e.g. [1,2,4,20]). In addition, there have been many attempts to model the morning-glory phenomenon based on existing nonlinear wave equations, such as the Korteweg–de Vries or Benjamin–Ono equations—see [21,22] for a discussion of geophysical flows for which these equations have been adopted as the phenomenological model of choice. To go beyond the range of validity of these models one typically argues, by analogy, that what is observed and measured appears to follow a familiar pattern and so some perturbation of a well-known equation might be relied upon to elucidate the essential features of the observations. The exercise is then one of fitting length scales, timescales and available coefficients in order to reproduce a particular wave structure; see, for example, [3,7,23,24]. In none of these discussions is a suitable equation derived from first principles, i.e. from the underlying, governing equations that describe the atmosphere. Of course, deriving such an equation from the general governing equations is a daunting undertaking, but crucial for a proper understanding of these flows. Here, we attempt to extract the main ingredients that contribute to the description of the underlying structure of waves of this type—and there is a bonus: we are able to be precise about the approximations needed to accomplish this and to describe the underlying temperature and pressure fields, as well as the heat sources required to drive and maintain the motion. Nevertheless, it must be emphasized that our mathematical approach, although far more general than hitherto attempted, does not easily reveal all the fine detail that we would wish. More analysis will be required, possibly linked to an extensive numerical investigation.

## 3. The governing equations

Since the governing equations for atmospheric flow are analytically intractable, careful approximations and simplifications are necessary, these providing the only realistic way to obtain useful and reliable analytical results. With this in mind, we advocate the use of systematic asymptotic expansions applied to the governing equations rather than starting from a model system or a model (nonlinear wave) equation. This is a significant improvement on most weather forecasting and climate models that are based on the hydrostatic primitive equations, with an underlying approximation which assumes a precise balance between the pressure and density fields within the framework of the ‘traditional approximation’. This simplification neglects the Coriolis terms that involve the cosine of the latitude for a thin-shell atmosphere. There is a rich analytic theory for the existence of solutions to these primitive equations (see the survey [25]). However, the hydrostatic approximation—a building block for the primitive equations—is not appropriate for a neutrally stratified atmosphere (see the discussion in [26]), which is the typical state near the top of a thermal inversion layer. Because our approach differs markedly from the usual one adopted for these type of studies, and to aid the reader less familiar with a development from first principles, we provide all the relevant details here (avoiding the need for the reader to access other similar, but different, analyses). We now present the formulation of the problem that underpins our discussion of nonlinear wave propagation in the atmosphere.

**Figure 4.**
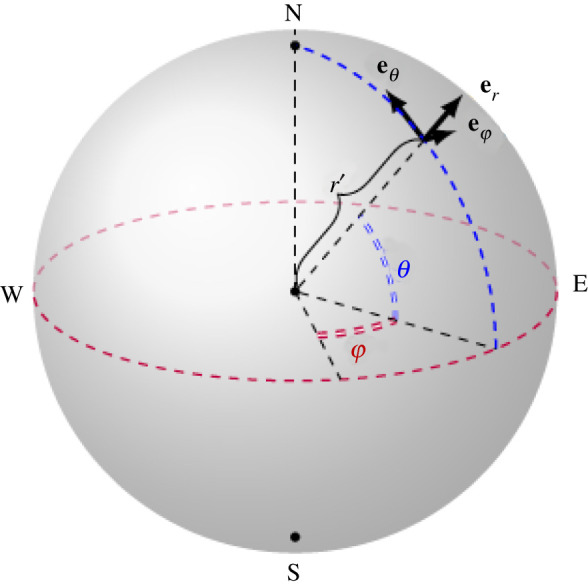
The spherical coordinate system $\left(\varphi ,\theta ,r^{\prime }\right)$, where θ is latitude, φ longitude and $r^{\prime }$ the distance from the origin at the Earth’s centre, is adequate to describe atmospheric flows outside polar regions. (Online version in colour.)

Throughout this analysis, we will assume a spherical Earth as the adjustments required to describe the oblateness are unimportant for this study of wave propagation; see [8,9] for details about the Earth’s geoid and how to model it. The coordinate system is the set of (right-handed) spherical coordinates $\left(\varphi ,\theta ,r^{\prime }\right)$: $r^{\prime }$ is the distance from the centre, $\theta \in [-\frac{\pi }{2},\frac{\pi }{2}]$ is the angle of latitude and φ∈[0,2π) is the azimuthal angle, i.e. the angle of longitude. The unit vectors in this system are $\left(e_{\varphi },\;e_{\theta },\;e_{r}\right)$ and the corresponding velocity components are $\left(u^{\prime },v^{\prime },w^{\prime }\right)$: $e_{\varphi }$ points from West to East, $e_{\theta }$ from South to North and $e_{r}$ points upwards (figure 4). The $\left(\varphi ,\theta ,r^{\prime }\right)$-system is associated with a point fixed on the sphere (other than at the two poles where the unit vectors are not well-defined) that is rotating about its polar axis (with constant angular speed $\Omega^{\prime }\approx 7.29\times 10^{-5}\;{\mathrm{rad}\;s}^{-1}$). At this stage, we use primes to denote dimensional (physical) variables; the primes will be removed when we introduce appropriate non-dimensional variables.

The Navier–Stokes equation, written in rotating spherical coordinates, is

3.1 $$\begin{array}{rl} & \rho^{\prime }\;\frac{\text{D}\;\;\;}{\text{D}t^{\prime }}\;(u^{\prime },v^{\prime },w^{\prime })+\frac{\rho^{\prime }}{r^{\prime }}(-u^{\prime }v^{\prime }\tan\theta +u^{\prime }w^{\prime },u^{\prime 2}\tan\theta +v^{\prime }w^{\prime },-u^{\prime 2}-v^{\prime 2})\\ & \;\;+2\Omega^{\prime }\rho^{\prime }\;(-\;v^{\prime }\sin\theta +w^{\prime }\cos\theta ,u^{\prime }\sin\theta ,-u^{\prime }\cos\theta )\\ & \;\;+r^{\prime }\Omega^{\prime 2}\rho^{\prime }\;(0,\;\sin\theta \cos\theta ,-\cos^{2}\theta )\\ & \;\;=-\nabla^{\prime }p^{\prime }+\rho^{\prime }(0,0,-g^{\prime }\;\frac{R^{\prime 2}}{r^{\prime 2}})+\nabla_{\mu }^{\prime 2}\;(u^{\prime },v^{\prime },w^{\prime })\\ & \;\;-\frac{2}{3}\;(\frac{1}{r^{\prime }\cos\theta }\;\frac{\partial }{\partial \varphi }(\frac{\mu_{h}^{\prime }}{\rho^{\prime }}\;\frac{D\rho^{\prime }}{Dt^{\prime }}),\frac{1}{r^{\prime }}\;\frac{\partial }{\partial \theta }(\frac{\mu_{h}^{\prime }}{\rho^{\prime }}\;\frac{D\rho^{\prime }}{Dt^{\prime }}),\frac{\partial }{\partial r^{\prime }}(\frac{\mu_{\nu }^{\prime }}{\rho^{\prime }}\;\frac{D\rho^{\prime }}{Dt^{\prime }}))\\ & \;\;-\frac{1}{r^{\prime 2}\cos^{2}\theta }\;(\mu_{h}^{\prime }u^{\prime },\;\mu_{h}^{\prime }v^{\prime },2\mu_{\nu }^{\prime }w^{\prime }(\cos^{2}\theta -v^{\prime }\sin\theta \;\cos\theta ))+\frac{2\mu_{h}^{\prime }}{r^{\prime 2}}\;\frac{\partial }{\partial \theta }\;(0,w^{\prime },-v^{\prime })\\ & \;\;+\frac{2\mu_{h}^{\prime }}{r^{\prime 2}\cos\theta }\;\frac{\partial }{\partial \varphi }\;(w^{\prime }-v^{\prime }\tan\theta ,\;u^{\prime }\tan\theta ,\;-u^{\prime })+\frac{\text{d}\mu_{\nu }^{\prime }}{\text{d}r^{\prime }}\;r^{\prime }\;\frac{\partial }{\partial r^{\prime }}(\frac{u^{\prime }}{r^{\prime }},\;\frac{v^{\prime }}{r^{\prime }},\;\frac{w^{\prime }}{r^{\prime }})\\ & \;\;+\frac{1}{r^{\prime }}\;(\frac{1}{r^{\prime }\cos\theta }\;\frac{\text{d}\mu_{h}^{\prime }}{\text{d}r^{\prime }}\;\frac{\partial }{\partial \varphi },\;\frac{1}{r^{\prime }}\;\frac{\text{d}\mu_{h}^{\prime }}{\text{d}r^{\prime }}\;\frac{\partial }{\partial \theta },\;\frac{\text{d}\mu_{\nu }^{\prime }}{\text{d}r^{\prime }}\;\frac{\partial }{\partial r^{\prime }})(r^{\prime }w^{\prime }), \end{array}$$

where $t^{\prime }$ is the time, $p^{\prime }(\varphi ,\theta ,r^{\prime },t^{\prime })$ the pressure, $\rho^{\prime }(\varphi ,\theta ,r^{\prime },t^{\prime })$ the density, and

3.2 $$\frac{\text{D}\;\;\;}{\text{D}t^{\prime }}\equiv (\frac{\partial }{\partial t^{\prime }}+\frac{u^{\prime }}{r^{\prime }\cos\theta }\frac{\partial }{\partial \varphi }+\frac{v^{\prime }}{r^{\prime }}\frac{\partial }{\partial \theta }+w^{\prime }\frac{\partial }{\partial r^{\prime }});\;\nabla^{\prime }\equiv (\frac{1}{r^{\prime }\cos\theta }\;\frac{\partial }{\partial \varphi },\;\frac{1}{r^{\prime }}\;\frac{\partial }{\partial \theta },\;\frac{\partial }{\partial r^{\prime }})$$

and

3.3 $$\nabla_{\mu }^{\prime 2}\equiv \mu_{\nu }^{\prime }(\frac{\partial^{2}}{\partial r^{\prime 2}}+\frac{2}{r^{\prime }}\;\frac{\partial }{\partial r^{\prime }})+\frac{\mu_{h}^{\prime }}{r^{\prime 2}}(\frac{1}{\cos^{2}\theta }\;\frac{\partial^{2}}{\partial \varphi^{2}}+\frac{\partial^{2}}{\partial \theta^{2}}-\tan\theta \frac{\partial }{\partial \theta }).$$

The gravitational body force in (3.1) is written in terms of $g^{\prime }\approx 9.81\;{m\;s}^{-2}$, the average acceleration of gravity at the surface of the Earth, of radius $R^{\prime }\approx 6371\;\mathrm{km}$, and we have introduced both coefficients of dynamic eddy viscosity ($\mu_{\nu }^{\prime }$ vertical and $\mu_{h}^{\prime }$ horizontal, each being a function of only $r^{\prime }$). In addition, we have incorporated Stokes’ hypothesis, namely, that the bulk viscosity coefficient is zero or, equivalently, the thermodynamic and mechanical pressures are equal; see [27].

The equation of mass conservation is

3.4 $$\frac{\text{D}\rho^{\prime }}{\text{D}t^{\prime }}+\rho^{\prime }\{\frac{1}{r^{\prime }\cos\theta }\;\frac{\partial u^{\prime }}{\partial \varphi }+\frac{1}{r^{\prime }\cos\theta }\;\frac{\partial }{\partial \theta }\;(v^{\prime }\cos\theta )+\frac{1}{r^{\prime 2}}\;\frac{\partial }{\partial r^{\prime }}\;(r^{\prime 2}w^{\prime })\}=0,$$

and the air is described by the equation of state for an ideal gas

3.5 $$p^{\prime }=\rho^{\prime }R^{\prime }T^{\prime },$$

$T^{\prime }$ being the absolute temperature (in K) and $R^{\prime }\approx 287$ m${\;}^{2}$ s${\;}^{-2}$ K${\;}^{-1}$ the gas constant. Together with this we have a suitable version of the first law of thermodynamics:

3.6 $$c_{p}^{\prime }\;\frac{\text{D}T^{\prime }}{\text{D}t^{\prime }}-\kappa^{\prime }\nabla^{\prime 2}T^{\prime }-\frac{1}{\rho^{\prime }}\;\frac{\text{D}p^{\prime }}{\text{D}t^{\prime }}=Q^{\prime }(\varphi ,\theta ,r^{\prime },t^{\prime }),$$

with $c_{p}^{\prime }\approx 10^{3}\;m^{2}\;s^{-2}\;K^{-1}$ the constant specific heat of predominantly dry air, $\kappa^{\prime }/c_{p}^{\prime }\approx 2\times 10^{-5}\;m^{2}\;s^{-1}$ the constant thermal diffusivity and $Q^{\prime }$ a general heat-source term; here

3.7 $$\nabla^{\prime 2}\equiv \frac{\partial^{2}}{\partial r^{\prime 2}}+\frac{2}{r^{\prime }}\;\frac{\partial }{\partial r^{\prime }}+\frac{1}{r^{\prime 2}}(\frac{1}{\cos^{2}\theta }\;\frac{\partial^{2}}{\partial \varphi^{2}}+\frac{\partial^{2}}{\partial \theta^{2}}-\tan\theta \frac{\partial }{\partial \theta }).$$

The second law of thermodynamics sets limits on the transformation between heat energy and the total mechanical energy, but this property will not directly impinge on the developments that we present here. (A more expansive description of this model for the atmosphere can be found in [8].) Finally, it is instructive to compute the vorticity associated with these flows: the vorticity vector, written in our spherical coordinates, is

3.8 $$\begin{array}{rl} \gamma^{\prime } & \;=(\frac{1}{r^{\prime }}\;\frac{\partial w^{\prime }}{\partial \theta }-\frac{\partial v^{\prime }}{\partial r^{\prime }}-\frac{v^{\prime }}{r^{\prime }})\;e_{\varphi }+(\frac{\partial u^{\prime }}{\partial r^{\prime }}+\frac{u^{\prime }}{r^{\prime }}-\frac{1}{r^{\prime }\cos\theta }\;\frac{\partial w^{\prime }}{\partial \varphi })\;e_{\theta }+\frac{1}{r^{\prime }}\;(u^{\prime }\tan\theta -\frac{\partial u^{\prime }}{\partial \theta }+\frac{1}{\cos\theta }\;\frac{\partial v^{\prime }}{\partial \varphi })\;e_{r}. \end{array}$$

In order to proceed, with the aim of extracting the relevant information from this general set of equations, we must non-dimensionalize and then introduce suitable parameters, together with an associated scaling and limiting process.

## 4. Non-dimensionalization and scaling

The non-dimensionalization is based on the timescale $1/\Omega^{\prime }$ (which is equivalent to about 3.5 hours—an appropriate choice for these wave motions) and on the speed scale $\Omega^{\prime }H^{\prime }$, where $H^{\prime }$ is a measure of the thickness of the troposphere (taken to be the maximum height of the troposphere—about 16 km). In addition, we require an average density of the air, ${\bar{\rho }}^{\prime }$, and an average dynamic eddy viscosity, ${\bar{\mu }}^{\prime }$; the fundamental parameter that we work with is $\varepsilon =H^{\prime }/R^{\prime }$, measuring the thinness of the atmospheric shell enveloping the Earth: we invoke the thin-shell approximation described by ε→0.

The non-dimensional (or normalized) variables—the unprimed variables—are then defined by

4.1 $$\left\{ \begin{array}{l} r^{\prime }=R^{\prime }(1+\varepsilon z),\;t=\Omega^{\prime }\;t^{\prime },\;\rho^{\prime }={\bar{\rho }}^{\prime }\rho ,\;p^{\prime }={\bar{\rho }}^{\prime }{\left(\Omega^{\prime }R^{\prime }\right)}^{2}p,\\ (u^{\prime },v^{\prime },w^{\prime })=\Omega^{\prime }H^{\prime }(u,v,w),\;T^{\prime }=\frac{{\left(\Omega^{\prime }R^{\prime }\right)}^{2}}{R^{\prime }}\;T. \end{array}\right.$$

We also introduce a number of (non-dimensional) parameters:

4.2 $$R_{e}=\frac{{\bar{\rho }}^{\prime }\Omega^{\prime }H^{\prime 2}}{{\bar{\mu }}^{\prime }},\;g=\frac{g^{\prime }H^{\prime }}{\Omega^{\prime 2}R^{\prime 2}},\;c_{p}=\frac{c_{p}^{\prime }}{R^{\prime }},\;\kappa =\frac{\kappa^{\prime }}{R^{\prime }\Omega^{\prime }H^{\prime 2}},$$

with

$$\omega =\frac{\Omega^{\prime }R^{\prime }}{U^{\prime }}\;(=\varepsilon^{-1}\;\text{for}\;U^{\prime }=\Omega^{\prime }H^{\prime }),$$

i.e. we have a small Rossby number. This condition on the Rossby number does not play a role in the standard modelling for waves such as the morning glory, but it is essential here in order to produce a consistent background state which we can then perturb. The parameters (4.2) are held fixed under the limiting process ε→0, so their values are immaterial; for information, the Reynolds number is $R_{e}\approx 10^{5}$, the thermal-conductivity parameter is $\kappa \approx 10^{-7}$, $c_{p}\approx 5.5$, g≈0.72. Even though two of these parameters take rather extreme values, the asymptotic procedure ensures that all the necessary physical attributes are retained. The guiding principle in this approach is: do not approximate unless absolutely necessary. (We could introduce thin viscous and thermal boundary layers, on the basis of these parameter values, but that is an additional mathematical complication which is avoided altogether in our approach. Any boundary layers are automatically included in our solution.)

We now non-dimensionalize equations (3.4), (3.5), (3.6) and (3.1), but we will write down only the leading-order terms, having invoked the thin-shell approximation (ε→0) in the viscous terms and noting the size of the relevant error terms. The Navier–Stokes equation becomes

4.3 $$\begin{array}{rl} & \rho \;\frac{\text{D}\;}{\text{D}t}\;(u,v,w)+\frac{\varepsilon \rho }{1+\varepsilon z}(-uv\tan\theta +uw,\;u^{2}\tan\theta +vw,-u^{2}-v^{2})\\ & \;\;+2\rho \;(-v\sin\theta +w\cos\theta ,\;u\sin\theta ,\;-u\cos\theta )+\frac{\rho (1+\varepsilon z)}{\varepsilon }\;(0,\;\sin\theta \cos\theta ,-\cos^{2}\theta )\\ & \;\;=-\frac{1}{\varepsilon^{2}}\;\nabla p+\frac{\rho }{\varepsilon^{2}}\;(0,0,-\frac{g}{{\left(1+\varepsilon z\right)}^{2}})\\ & \;\;+\frac{1}{R_{e}}\;\{(\frac{\partial }{\partial z}[m(z)\;\frac{\partial }{\partial z}]+M(z)\;[\frac{\partial^{2}}{\partial {\left(\theta /\varepsilon \right)}^{2}}+\frac{1}{\cos^{2}\theta }\;\frac{\partial^{2}}{\partial {\left(\varphi /\varepsilon \right)}^{2}}])(u,v,w)+O(\varepsilon )\}, \end{array}$$

where

4.4 $$\frac{\text{D}\;\;}{\text{D}t}\equiv (\frac{\partial }{\partial t}+\varepsilon \;[\frac{u}{(1+\varepsilon z)\cos\theta }\frac{\partial }{\partial \varphi }+\frac{v}{1+\varepsilon z}\frac{\partial }{\partial \theta }+\frac{w}{\varepsilon }\;\frac{\partial }{\partial z})$$

and

4.5 $$\nabla \equiv (\frac{\varepsilon }{(1+\varepsilon z)\cos\theta }\frac{\partial }{\partial \varphi },\frac{\varepsilon }{1+\varepsilon z}\frac{\partial }{\partial \theta }\;\frac{\partial }{\partial z}),$$

and we have written the two eddy viscosities as

$$\mu_{\nu }^{\prime }={\bar{\mu }}^{\prime }\;m(z),\;\mu_{h}^{\prime }={\bar{\mu }}^{\prime }\;M(z).$$

Correspondingly, the equation of mass conservation becomes

4.6 $$\frac{\text{D}\rho }{\text{D}t}+\rho \{\frac{\varepsilon }{(1+\varepsilon z)\cos\theta }(\frac{\partial u}{\partial t}+\frac{\partial }{\partial \theta }\;(v\cos\theta ))+\frac{1}{{\left(1+\varepsilon z\right)}^{2}}\;\frac{\partial }{\partial z}\;[{\left(1+\varepsilon z\right)}^{2}w]\}=0,$$

and the equation of state is

4.7 p=ρT;

the first law of thermodynamics is written as

4.8 $$c_{p}\;\frac{\text{D}T}{\text{D}t}-\frac{1}{\rho }\;\frac{\text{D}\rho }{\text{D}t}-\kappa \;\nabla^{2}T=Q(\varphi ,\theta ,z,t;\varepsilon ),$$

where

4.9 $$\nabla^{2}\equiv \frac{\partial^{2}}{\partial z^{2}}+\frac{2\varepsilon }{1+\varepsilon z}\;\frac{\partial }{\partial z}+\frac{\varepsilon^{2}}{{\left(1+\varepsilon z\right)}^{2}}(\frac{1}{\cos^{2}\theta }\;\frac{\partial^{2}}{\partial \varphi^{2}}+\frac{\partial^{2}}{\partial \theta^{2}}-\tan\theta \;\frac{\partial }{\partial \theta }),$$

and the non-dimensional heat-source term is Q. The corresponding form of the vorticity is

4.10 $$\begin{array}{rl} \gamma & \;=(\frac{\varepsilon }{1+\varepsilon z}\;\frac{\partial w}{\partial \theta }-\frac{\partial v}{\partial z}-\frac{\varepsilon v}{1+\varepsilon z})\;e_{\varphi }+(\frac{\partial u}{\partial z}+\frac{\varepsilon u}{1+\varepsilon z}-\frac{\varepsilon }{(1+\varepsilon z)\cos\theta }\;\frac{\partial w}{\partial \varphi })\;e_{\theta }\\ & \;\;+\frac{\varepsilon }{1+\varepsilon z}\;(u\tan\theta -\frac{\partial u}{\partial \theta }+\frac{1}{\cos\theta }\;\frac{\partial v}{\partial \varphi })\;e_{r}, \end{array}$$

where $\gamma^{\prime }=\gamma \Omega^{\prime }$. This system of equations corresponds closely to that used to study the properties of the steady atmosphere [8], and various large-scale wave motions [9], but there are important differences. Although the timescale used here and in [9] is the same, the size of the velocity component in the vertical direction here, as compared with that used in [8,9], differs; this leads to a very significant change in the structure of the solution. This arises mainly because of the requirement here to scale the variations in the direction of propagation, and along the wavefront, differently. This also leads to a slightly different form of the background state of the atmosphere, onto which the motion is superimposed. Further, in order to apply these equations to nonlinear wave phenomena (such as the morning glory), we must describe a wave propagating in a specific direction, with little variation along the wavefront and a stronger variation in the direction of propagation.

To initiate this further development, we note that waves of the morning-glory type, for example, as seen in a number of locations around the world, can extend up to 3 km or so above the Earth’s surface, but rarely is the wave train as long as 30 km; indeed, individual rolls are typically only a few hundred metres in diameter. The length of the wavefront is usually well over 100 km and, on occasions, it is observed to be as much as 1000 km, with the whole wave group sometimes propagating over hundreds of kilometres [10,12]; on these length scales, it is not advisable to ignore the effects of spherical geometry. We proceed by using the thin-shell parameter, ε, to provide a scale that differentiates the structure in the propagation direction from that along the wavefront. First, however, we must introduce a horizontal rotation of the coordinate system about a point on the surface of the Earth.

We rotate (φ,θ) through the fixed angle α, counter-clockwise about the fixed point $\left(\varphi_{0},\theta_{0}\right)$, to produce (Φ,Θ); the wave is propagating in the Θ-direction (and we might expect weak variation along the wavefront, i.e. in the Φ-direction, presumably coupled with no flow in this direction). Thus, we have

$$\varphi -\varphi_{0}=\Phi \cos\alpha -\Theta \sin\alpha ,\;\theta -\theta_{0}=\Phi \sin\alpha +\Theta \cos\alpha ,$$

with the corresponding velocity components (U,V), where

4.11 $$\begin{array}{rl} & u=U\cos\alpha -V\sin\alpha \\ \;\mathrm{and}\; & v=U\sin\alpha +V\cos\alpha . \end{array}\}$$

Further, we require that the scale associated with the Θ-direction is much less than that in the Φ-direction; indeed, in terms of the formulation that we have presented, the natural choice is to replace Θ by εΘ. Thus, the transformation that we use is

4.12 $$\varphi -\varphi_{0}=\Phi \cos\alpha -\varepsilon \;\Theta \sin\alpha \;\mathrm{and}\;\theta -\theta_{0}=\Phi \sin\alpha +\varepsilon \;\Theta \cos\alpha ,$$

in conjunction with (4.11). The description that we propose, therefore, is that the scaling in the direction of propagation is comparable with that in the vertical direction, i.e. the depth of the troposphere guides the length of the wave train; the length of the wavefront, on this scale, is then Φ=O(1). For comparison, we can note, with $H^{\prime }=15\;\text{km}$, that a wave with its top at 3 km corresponds to z=1/5, and a length of the wave train of 30 km corresponds to Θ=2 (ignoring the adjustment from α): both are reasonable O(1) choices. More significantly, this scaling ensures, at the order at which the leading-order velocity field is determined, that we have a consistent nonlinear system that is derived directly from the general governing equations.

We use (4.11) and (4.12) in equations (4.6) and (4.3), with each component written out to give

4.13 $$\frac{\hat{\text{D}}\rho }{\text{D}t}+\rho \{(1-\frac{1}{\hat{C}})\sin\alpha \cos\alpha \;\frac{\partial U}{\partial \Theta }+(\cos^{2}\alpha +\frac{\sin^{2}\alpha }{\hat{C}})\;\frac{\partial V}{\partial \Theta }+\frac{\partial w}{\partial z}\}=O(\varepsilon ),$$

4.14 $$\begin{array}{rl} & \rho \;\frac{\hat{\text{D}}\;}{\text{D}t}\;(U\cos\alpha -V\sin\alpha )-2\rho (U\sin\alpha +V\cos\alpha )\hat{S}+2\rho w\hat{C}\\ & \;=-\frac{1}{\varepsilon^{2}(1+\varepsilon z)\hat{C}}\;(\varepsilon \;\frac{\partial p}{\partial \Phi }\;\cos\alpha -\frac{\partial p}{\partial \Theta }\;\sin\alpha )\\ & \;\;+\frac{1}{R_{e}}\;\{(\frac{\partial }{\partial z}[m(z)\;\frac{\partial }{\partial z}]+M(z)\;[\cos^{2}\alpha +\frac{\sin^{2}\alpha }{{\hat{C}}^{2}}]\;\frac{\partial^{2}}{\partial \Theta^{2}}\}\;(U\cos\alpha -V\sin\alpha )+O(\varepsilon ), \end{array}$$

4.15 $$\begin{array}{rl} & \rho \;\frac{\hat{\text{D}}\;}{\text{D}t}\;(U\sin\alpha +V\cos\alpha )+2\rho (U\cos\alpha -V\sin\alpha )\hat{S}+\frac{1}{\varepsilon }\;\rho (1+\varepsilon z)\hat{S}\hat{C}\\ & \;=-\frac{1}{\varepsilon^{2}(1+\varepsilon z)}\;(\frac{\partial p}{\partial \Theta }\;\cos\alpha +\varepsilon \;\frac{\partial p}{\partial \Phi }\;\sin\alpha )\\ & \;\;+\frac{1}{R_{e}}\;\{(\frac{\partial }{\partial z}[m(z)\;\frac{\partial }{\partial z}]+M(z)[\cos^{2}\alpha +\frac{\sin^{2}\alpha }{{\hat{C}}^{2}}]\;\frac{\partial^{2}}{\partial \Theta^{2}}\}\;(U\sin\alpha +V\cos\alpha )+O(\varepsilon ), \end{array}$$

4.16 $$\begin{array}{rl} & \rho \;\frac{\hat{\text{D}}w}{\text{D}t}-2\rho (U\cos\alpha -V\sin\alpha )\hat{C}-\frac{1}{\varepsilon }\;\rho (1+\varepsilon z){\hat{C}}^{2}=-\frac{1}{\varepsilon^{2}}\;\{\frac{\partial p}{\partial z}+\frac{\rho g}{{\left(1+\varepsilon z\right)}^{2}}\}+O(\varepsilon ), \end{array}$$

where

$$\begin{array}{rl} & \frac{\hat{\text{D}}\;}{\text{D}t}=\frac{\partial }{\partial t}+\frac{1}{1+\varepsilon z}\{U(1-\frac{1}{\hat{C}})\sin\alpha \cos\alpha +V(\cos^{2}\alpha +\frac{\sin^{2}\alpha }{\hat{C}})\}\;\frac{\partial }{\partial \Theta }\\ & \;\;+\frac{\varepsilon }{1+\varepsilon z}\{U(\sin^{2}\alpha +\frac{\cos^{2}\alpha }{\hat{C}})+V(1-\frac{1}{\hat{C}})\sin\alpha \cos\alpha \}\;\frac{\partial }{\partial \Phi }+w\;\frac{\partial }{\partial z} \end{array}$$

and

$$\hat{S}=\sin(\theta_{0}+\Phi \sin\alpha +\varepsilon \;\Theta \cos\alpha )\;\mathrm{and}\;\hat{C}=\cos(\theta_{0}+\Phi \sin\alpha +\varepsilon \;\Theta \cos\alpha ).$$

In addition, we have (4.7) and, from equation (4.8),

4.17 $$c_{p}\;\frac{\hat{\text{D}}T}{\text{D}t}-\frac{1}{\rho }\;\frac{\hat{\text{D}}\rho }{\text{D}t}-\kappa \;{\hat{\nabla }}^{2}T=\hat{Q}(\Phi ,\Theta ,z,t;\;\varepsilon ),$$

where

4.18 $$\begin{array}{rl} {\hat{\nabla }}^{2} & \;\equiv \frac{\partial^{2}}{\partial z^{2}}+(\frac{\sin^{2}\alpha }{C^{2}}+\cos^{2}\alpha )\;\frac{\partial^{2}}{\partial \Theta^{2}}\\ & \;\;+2\varepsilon \{\frac{\partial }{\partial z}+(\Theta \;\frac{S}{C^{3}}\;\cos\alpha \sin^{2}\alpha -z\;\cos^{2}\alpha -z\;\frac{\sin^{2}\alpha }{C^{2}})\;\frac{\partial^{2}}{\partial \Theta^{2}}\\ & \;\;+(1-\frac{1}{C^{2}})\;\sin\alpha \cos\alpha \;\frac{\partial^{2}}{\partial \Phi \partial \Theta }-\frac{S}{2C}\;\cos\alpha \;\frac{\partial }{\partial \Theta }\}+O(\varepsilon^{2}), \end{array}$$

with

4.19 $$S=\sin(\theta_{0}+\Phi \sin\alpha )\;\mathrm{and}\;C=\cos(\theta_{0}+\Phi \sin\alpha ).$$

As we shall see, this appearance of Φ is only parametric in the wave-evolution equation, describing the distortion along the wavefront, consistent with using spherical coordinates. Finally, we compute the corresponding form of the vorticity following the rotation of the horizontal vectors; we find that

4.20 $$\begin{array}{rl} \gamma & \;=\frac{1}{1+\varepsilon z}\;\{-\varepsilon \sin\alpha \;\frac{S}{C}\;V+\varepsilon (\sin^{2}\alpha +\frac{\cos^{2}\alpha }{C})\;\frac{\partial V}{\partial \Phi }+(1-\frac{1}{C})\sin\alpha \cos\alpha \;\frac{\partial V}{\partial \Theta }\}\;e_{r}\\ & \;\;+\{\frac{\varepsilon \sin\alpha \cos\alpha }{1+\varepsilon z}(1-\frac{1}{C})\;\frac{\partial w}{\partial \Phi }+\frac{1}{1+\varepsilon z}\;(\cos^{2}\alpha +\frac{\sin^{2}\alpha }{C})\;\frac{\partial w}{\partial \Theta }-\frac{\partial V}{\partial z}-\frac{\varepsilon V}{1+\varepsilon z}\}\;e_{f}\\ & \;\;+\{-\;\frac{\varepsilon }{1+\varepsilon z}(\sin^{2}\alpha +\frac{\cos^{2}\alpha }{C})\;\frac{\partial w}{\partial \Phi }+\frac{\sin\alpha \cos\alpha }{1+\varepsilon z}(\frac{1}{C}-1)\;\frac{\partial w}{\partial \Theta }\}\;e_{d}+O(\varepsilon^{2}), \end{array}$$

where ${\text{e}}_{d}$ is the unit vector in the direction of propagation, and ${\text{e}}_{f}$ the unit vector along the wavefront, directed such that the orthonormal basis $\left\{{\text{e}}_{d},\;{\text{e}}_{r},\;{\text{e}}_{f}\right\}$ is positively oriented. We now construct the asymptotic solution of equations (4.7), (4.13)–(4.17).

## 5. Asymptotic structure of the equations

We seek a solution of our transformed governing equations, (4.7), (4.13)–(4.17), by expanding all the coefficients (and $\hat{Q}$) in powers of ε, and then writing

$$q(\Phi ,\Theta ,z,t,\varepsilon )∼\sum_{n=0}^{\infty }\varepsilon^{n}q_{n}(\Phi ,\Theta ,z,t),$$

where q (and correspondingly $q_{n}$) represent each of the variables p, ρ, T, U, V, w. The asymptotic solution is valid only for the three independent variables (Φ,Θ,z) each to be O(1); indeed, this choice is controlled by the existence of the troposphere (whose top is, at most, at z=1), and we aim to find solutions which have suitable decay conditions at each end of the wave train and at the extremities of the wavefront. The development of the solution proceeds in two stages: first, we find the leading order and O(ε) that describe the background state of the atmosphere, and then the leading-order velocity field, which is a nonlinear problem, is determined at $\text{O}(\varepsilon^{2})$; we start with the first two orders.

At O(1), i.e. the leading terms in the asymptotic expansion of the solution, we have

5.1 $$\begin{array}{r} \frac{\partial p_{0}}{\partial \Theta }=0,\;\frac{\partial p_{0}}{\partial z}=-g\rho_{0},\;p_{0}=\rho_{0}T_{0}\;, \end{array}$$

5.2 $$\begin{array}{r} c_{p}\;D_{0}(T_{0})-\frac{1}{\rho_{0}}\;D_{0}(p_{0})-\kappa \;\nabla_{0}^{2}(T_{0})=Q_{0}, \end{array}$$

where

$$\begin{array}{rl} D_{0} & \;\equiv \frac{\partial }{\partial t}+\{U_{0}(1-\frac{1}{C})\sin\alpha \cos\alpha +V_{0}(\cos^{2}\alpha +\frac{\sin^{2}\alpha }{C})\}\;\frac{\partial }{\partial \Theta }+w_{0}\;\frac{\partial }{\partial z},\\ \nabla_{0}^{2} & \;\equiv \frac{\partial^{2}}{\partial z^{2}}+(\cos^{2}\alpha +\frac{\sin^{2}\alpha }{C})\;\frac{\partial^{2}}{\partial \Theta^{2}}. \end{array}$$

(Note that the first equation in the set (5.1)–(5.2) occurs twice.) The solution of this system will be addressed when we have obtained the Φ-dependence that appears in the leading-order system; this is generated by the equations at the next order.

We proceed to examine the O(ε) problem; this produces

5.3 $$\begin{array}{rl} & \sin\alpha \;\frac{\partial p_{1}}{\partial \Theta }=\cos\alpha \;\frac{\partial p_{0}}{\partial \Phi }, \end{array}$$

5.4 $$\begin{array}{rl} & \cos\alpha \;\frac{\partial p_{1}}{\partial \Theta }=-\sin\alpha \;\frac{\partial p_{0}}{\partial \Phi }-\rho_{0}\;SC, \end{array}$$

5.5 $$\begin{array}{rl} & \frac{\partial p_{1}}{\partial z}=-g\rho_{1}+[2gz+C^{2}]\;\rho_{0}, \end{array}$$

5.6 $$\begin{array}{rl} & p_{1}=\rho_{0}T_{1}+\rho_{1}T_{0},\\ & c_{p}\;\{D_{0}(T_{1})+D_{1}(T_{0})\}-\frac{1}{\rho_{0}}\;\{D_{0}(p_{1})+D_{1}(p_{0})-\frac{\rho_{1}}{\rho_{0}}\;D_{0}(p_{0})\} \end{array}$$

5.7 $$\begin{array}{rl} \;\mathrm{and}\; & \;-\kappa \;\nabla_{0}^{2}(T_{1})-2\kappa \;\frac{\partial T_{0}}{\partial Z}=Q_{1}, \end{array}$$

where we have written

$$\frac{\hat{\text{D}}\;}{\text{D}t}\equiv D_{0}+\varepsilon \;D_{1}+O(\varepsilon^{2}).$$

Now (5.3) and (5.4) can be re-expressed as

5.8 $$\frac{\partial p_{0}}{\partial \Phi }=-\rho_{0}SC\sin\alpha \;,\;\frac{\partial p_{0}}{\partial \Theta }=-\rho_{0}SC\cos\alpha .$$

The first equation in (5.8), in conjunction with (5.1), shows that $p_{0}$, $\rho_{0}$ and $T_{0}$ are functions of

5.9 $$\zeta =gz+\frac{1}{2}\;S^{2},$$

with

5.10 $$\frac{dp_{0}}{d\zeta }=-\rho_{0}\;,\;p_{0}=\rho_{0}T_{0},$$

for some $T_{0}(\zeta )$. The first law of thermodynamics at this order, (5.2), gives

5.11 $$gw_{0}(c_{p}\;\frac{dT_{0}}{d\zeta }+1)-\kappa g^{2}\;\frac{d^{2}T_{0}}{d\zeta^{2}}=Q_{0}\;$$

and a solution for $Q_{0}\equiv 0$ is

5.12 $$T_{0}(\zeta )=T_{0}(0)-\frac{\zeta }{c_{p}},$$

where $T_{0}(0)$ is an arbitrary constant. This is the familiar solution that describes the ambient state of the troposphere (independent of the velocity field), with temperature decreasing linearly upwards [8]. Further, because this solution corresponds to $Q_{0}\equiv 0$, no external heat sources are required, at this order. However, as we will see later, other solutions of (5.11) must be considered if we aim to provide a reasonable basis for the generation of waves such as the morning glory; see §6b.

Returning to equation (5.7), we find that it can be written as

5.13 $$\begin{array}{rl} & c_{p}\;D_{0}(T_{1})-\frac{1}{\rho_{0}}\;D_{0}(p_{1})+\frac{\rho_{1}}{\rho_{0}^{2}}\;\frac{\partial p_{0}}{\partial z}\\ & \;\;+\{U_{0}(\sin^{2}\alpha +\frac{\cos^{2}\alpha }{C})+V_{0}(1-\frac{1}{C})\sin\alpha \cos\alpha \}(c_{p}\;\frac{\partial T_{0}}{\partial \Phi }+SC\sin\alpha )\\ & \;\;+w_{1}(c_{p}\;\frac{\partial T_{0}}{\partial z}+g)-\kappa \;\nabla_{0}^{2}(T_{1})=Q_{1}+2\kappa \;\frac{\partial T_{0}}{\partial z}, \end{array}$$

which can be used to determine the heat sources at this order, given the background thermodynamic state and the velocity field $\left(U_{0},V_{0},w_{0}\right)$ and $w_{1}$.

Eliminating $p_{1}$ between (5.5) and (5.6), and setting

5.14 $$T_{1}=T_{0}^{2}\;\frac{\partial \tau_{1}}{\partial z},$$

we find that

5.15 $$p_{1}=p_{0}\{g\tau_{1}+\int_{0}^{z}\frac{2gz+C^{2}}{T_{0}(gz^{\prime }+\frac{1}{2}\;S^{2})}\;dz^{\prime }+F(\Phi ,\Theta ,t)\},$$

and then the second equation of the pair in (5.8) gives

$$\tau_{1}=-\frac{SC\cos\alpha }{g}\;\frac{\Theta }{T_{0}}+G(\Phi ,z,t)-\frac{1}{g}\;F(\Phi ,\Theta ,t),$$

where F and G are arbitrary functions. We also have

5.16 $$\rho_{1}=g\rho_{0}G-p_{0}\;\frac{\partial G}{\partial z}+\rho_{0}\int_{0}^{z}\frac{2gz+C^{2}}{T_{0}(gz^{\prime }+\frac{1}{2}\;S^{2})}\;dz^{\prime }-\frac{\rho_{0}}{T_{0}}\;SC\;\Theta \;(1+\frac{dT_{0}}{d\zeta })\cos\alpha$$

and

5.17 $$T_{1}=\frac{SC\cos\alpha }{g}\;\Theta \;\frac{\partial T_{0}}{\partial z}+T_{0}^{2}\;\frac{\partial G}{\partial z}.$$

Combining all the above results, the first law, (5.13), which describes the internal heating and heat sources for this flow at this order, can be written as

5.18 $$\begin{array}{rl} & T_{0}\;\frac{\partial }{\partial t}(c_{p}T_{0}\;\frac{\partial G}{\partial z}-gG)+w_{0}\{c_{p}\;\frac{\partial }{\partial z}(T_{0}^{2}\frac{\partial G}{\partial z})+g(T_{0}\;\frac{\partial G}{\partial z}-gG)-C^{2}-2gz\}\\ & \;\;+\{U_{0}(\sin^{2}\alpha +\frac{\cos^{2}\alpha }{C})+V_{0}(1-\frac{1}{C})\sin\alpha \cos\alpha \}(c_{p}\;\frac{\partial T_{0}}{\partial \Phi }+SC\sin\alpha )\\ & \;\;+w_{1}(c_{p}\;\frac{\partial T_{0}}{\partial z}+g)-\kappa \;\frac{\partial^{2}}{\partial z^{2}}(T_{0}^{2}\;\frac{\partial G}{\partial z})=Q_{1}+2\kappa \;\frac{\partial T_{0}}{\partial z}\;. \end{array}$$

This completes the description of the background state, correct to O(ε), which permits, at this stage, some choices via the arbitrary functions F and G. In our approach to the problem, we treat (5.18) as an expression for $Q_{1}$, enabling us to identify the heat sources required to drive and maintain the motion (at this order), given the velocity field at leading order, $\left(U_{0},V_{0},w_{0}\right)$, and $w_{1}$ from the next order. We note, however, that $U_{0}$, $V_{0}$ and $w_{1}$ are eliminated from this expression if $T_{0}$ is defined by (5.12). Further, we expect to be able to obtain sufficient information from the leading order alone, and so provide a reasonable description of the atmospheric processes involved.

At the next order, $\text{O}(\varepsilon^{2})$, we obtain the equations that govern the leading-order velocity field; it is the construction and interpretation of these that is the main thrust of this work. We perform the combination that led to equations (5.8), and so we are able to obtain the equation

5.19 $$\begin{array}{rl} \frac{\partial p_{1}}{\partial \Phi } & \;=-\rho_{0}D_{0}\{(\sin^{2}\alpha +C\;\cos^{2}\alpha )U_{0}+(1-C)V_{0}\sin\alpha \cos\alpha \}\\ & \;\;-2\rho_{0}S\{(1-C)U_{0}\sin\alpha \cos\alpha -(\sin^{2}\alpha +C\cos^{2}\alpha )V_{0}\}-2\rho_{0}C^{2}w_{0}\cos\alpha \\ & \;\;-\rho_{1}SC\sin\alpha -\rho_{0}\sin\alpha \{2z\;SC+\Theta \;(C^{2}-S^{2})\cos\alpha \}\\ & \;\;+\frac{1}{R_{e}}\;\Delta_{0}\{(\sin^{2}\alpha +C\cos^{2}\alpha )U_{0}+(1-C)V_{0}\sin\alpha \cos\alpha \}, \end{array}$$

where we have written

5.20 $$\Delta_{0}\equiv \frac{\partial }{\partial z}(m(z)\;\frac{\partial }{\partial z})+M(z)(\cos^{2}\alpha +\frac{\sin^{2}\alpha }{C})\;\frac{\partial^{2}}{\partial \Theta^{2}},$$

and we also have

5.21 $$D_{0}(\rho_{0})+\rho_{0}\{(1-\frac{1}{C})\sin\alpha \cos\alpha \;\frac{\partial U_{0}}{\partial \Theta }+(\cos^{2}\alpha +\frac{\sin^{2}\alpha }{C})\;\frac{\partial V_{0}}{\partial \Theta }+\frac{\partial w_{0}}{\partial z}\}=0.$$

Expressions for $\partial p_{2}/\partial \Theta$ and $\partial p_{2}/\partial z$ can also be found, but they are not needed for the determination of the background state at leading order, nor for the construction of the nonlinear wave equation; we would need to use them if we wish to find $w_{1}$. Also, the equation of state and the first law of thermodynamics, at this order, are not required for the description of the nonlinear wave motion, nor for the background state, as presented here.

We see that equations (5.19) and (5.21) relate the components of the velocity field $\left(U_{0},V_{0},w_{0}\right)$, knowing the background state to leading order. Once we have determined all these, we may then use this information in the first law of thermodynamics to provide an interpretation of the heat sources required to maintain the motion. Now, because (5.19) and (5.21) involve the three components of the velocity field, we may proceed by assuming that there is no flow along the wavefront: $U_{0}\equiv 0$. (We mentioned this possibility earlier, but have not invoked this condition thus far.) This then produces two equations for $\left(V_{0},w_{0}\right)$. So, firstly, equation (5.19) can be written as

5.22 $$\begin{array}{rl} \frac{\partial p_{1}}{\partial \Phi } & \;=-\rho_{0}(1-C)\sin\alpha \cos\alpha \;\bar{D_{0}}(V_{0})\\ & \;\;+2\rho_{0}S(\sin^{2}\alpha +C\cos^{2}\alpha )V_{0}-2\rho_{0}C^{2}w_{0}\cos\alpha -\rho_{1}SC\sin\alpha \\ & \;\;-\rho_{0}\{2z\;SC+\Theta \;(C^{2}-S^{2})\cos\alpha \}\sin\alpha +\frac{1}{R_{e}}\;(1-C)\sin\alpha \cos\alpha \;\Delta_{0}(V_{0}), \end{array}$$

where

$$\bar{D_{0}}\equiv \frac{\partial }{\partial t}+(\cos^{2}\alpha +\frac{\sin^{2}\alpha }{C})\;V_{0}\;\frac{\partial }{\partial \Theta }+w_{0}\;\frac{\partial }{\partial z},$$

and then, using (5.15), the forcing term

$$\frac{\partial p_{1}}{\partial \Phi }+\rho_{1}SC\sin\alpha +\rho_{0}\{2z\;SC+\Theta \;(C^{2}-S^{2})\cos\alpha \}\sin\alpha ,$$

can be expressed as

$$p_{0}(g\;\frac{\partial G}{\partial \Phi }-SC\sin\alpha \;\frac{\partial G}{\partial z})+\rho_{0}SC\sin\alpha \{4z+\frac{C^{2}}{g}(1+\frac{T_{0}}{T_{0}(0)})-4T_{0}\;\int_{0}^{z}\frac{dz^{\prime }}{T_{0}(gz^{\prime }+\frac{1}{2}\;S^{2}}\},$$

which we note does not depend on Θ. The component of the Navier–Stokes equation, (5.22), can then be written as

5.23 $$\begin{array}{rl} & \;(1-C)\sin\alpha \cos\alpha \;\bar{D_{0}}(V_{0})-2S(\sin^{2}\alpha +C\cos^{2}\alpha )V_{0}\\ & \;\;+2C^{2}w_{0}\cos\alpha -\frac{1}{R_{e}}\;(1-C)\sin\alpha \cos\alpha \;\Delta_{0}(V_{0})+T_{0}(g\;\frac{\partial G}{\partial \Phi }-SC\sin\alpha \;\frac{\partial G}{\partial z})\\ & \;\;+SC\sin\alpha \;\{4z+\frac{C^{2}}{g}(1+\frac{T_{0}}{T_{0}(0)})-4T_{0}\;\int_{0}^{z}\frac{dz^{\prime }}{T_{0}(gz^{\prime }+\frac{1}{2}\;S^{2})}\}=0. \end{array}$$

Correspondingly, the equation of mass conservation at this order, (5.21), reduces to

5.24 $$w_{0}\;\frac{\partial \rho_{0}}{\partial z}+\rho_{0}\{(\cos^{2}\alpha +\frac{\sin^{2}\alpha }{C})\;\frac{\partial V_{0}}{\partial \Theta }+\frac{\partial w_{0}}{\partial z}\}=0,$$

because $\rho_{0}=\rho_{0}(\Phi ,z)$.

We now examine equations (5.23) and (5.24) in a little more detail; for convenience we write (5.23) as

5.25 $$\begin{array}{rl} & \;(1-C)\sin\alpha \cos\alpha \;\bar{D_{0}}(V_{0})-2S(\sin^{2}\alpha +C\cos^{2}\alpha )V_{0}\\ & \;\;+2C^{2}w_{0}\cos\alpha -\frac{1}{\rho_{0}R_{e}}\;(1-C)\sin\alpha \cos\alpha \;\Delta_{0}(V_{0})=K(\Phi ,z,t), \end{array}$$

where the forcing term

5.26 $$\begin{array}{rl} & K(\Phi ,z,t)=-T_{0}(g\;\frac{\partial G}{\partial \Phi }-SC\sin\alpha \;\frac{\partial G}{\partial z})+SC\sin\alpha \;\{4z+\frac{C^{2}}{g}(1+\frac{T_{0}}{T_{0}(0)})-4T_{0}\;\int_{0}^{z}\frac{dz^{\prime }}{T_{0}(gz^{\prime }+\frac{1}{2}\;S^{2})}\}, \end{array}$$

can be regarded as arbitrary, because G is. If we elect to use a particular K, in order to produce a suitable solution, we may then identify G. Finally, we see that equation (5.24) leads to the introduction of a stream function, Ψ(Φ,Θ,z,t), with

5.27 $$(\cos^{2}\alpha +\frac{\sin^{2}\alpha }{C})\;\rho_{0}V_{0}=\frac{\partial \Psi }{\partial z},\;\rho_{0}w_{0}=-\frac{\partial \Psi }{\partial \Theta },$$

and then equation (5.25) becomes

5.28 $$\begin{array}{rl} & \frac{\partial^{2}\Psi }{\partial z\partial t}+\frac{1}{\rho_{0}}\;\frac{\partial \Psi }{\partial z}\;\frac{\partial^{2}\Psi }{\partial z\partial \Theta }-\frac{\partial \Psi }{\partial \Theta }\;\frac{\partial }{\partial z}(\frac{1}{\rho_{0}}\;\frac{\partial \Psi }{\partial z})\\ & \;\;-\sigma (S\;\frac{\partial \Psi }{\partial z}+C\cos\alpha \;\frac{\partial \Psi }{\partial \Theta })-\frac{1}{R_{e}}\;\Delta_{0}(\frac{1}{\rho_{0}}\;\frac{\partial \Psi }{\partial z})=K(\Phi ,z,t), \end{array}$$

where

5.29 $$\sigma =\frac{2(\sin^{2}\alpha +C\cos^{2}\alpha )}{(1-C)\sin\alpha \cos\alpha }.$$

This is our fundamental nonlinear equation, which describes the properties of the wave, given the background state of the atmosphere; we note that Φ appears only as a parameter in this equation, although its retention is important when we note that the wavefront can extend as much as 1000 km. Equation (5.28) describes the leading-order velocity field, i.e. the solution at O(1), even though the balance that produces it appears at $\text{O}(\varepsilon^{2})$. In addition, we are able to determine the vorticity associated with this solution; from (4.18), we see that the vorticity vector, at leading order, becomes

5.30 $$\begin{array}{rl} & \gamma_{0}=(1-\frac{1}{C})\sin\alpha \cos\alpha \;\frac{\partial V_{0}}{\partial \Theta }\;e_{r}+\{(\cos^{2}\alpha +\frac{\sin^{2}\alpha }{C})\;\frac{\partial w_{0}}{\partial \Theta }-\frac{\partial V_{0}}{\partial z}\}\;e_{f}\\ & \;\;+\sin\alpha \cos\alpha (\frac{1}{C}-1)\;\frac{\partial w_{0}}{\partial \Theta }\;e_{d}. \end{array}$$

Note that if we regard (5.28) as an evolution equation for a two-dimensional flow with velocity components $\left(V_{0},\;w_{0},\;0\right)$, dependent on the variables (X,z,Φ) with $X=\Theta /(\cos^{2}\alpha +\sin^{2}\alpha /C)$, with respect to the orthogonal frame $\left({\text{e}}_{d},\;{\text{e}}_{r},\;{\text{e}}_{f}\right)$, the corresponding curl of the velocity is

5.31 $$\frac{\partial V_{0}}{\partial \Phi }\;e_{r}+\{\frac{\partial w_{0}}{\partial X}-\frac{\partial V_{0}}{\partial z}\}\;e_{f}-\frac{\partial w_{0}}{\partial \Phi }\;e_{d}=\frac{\partial V_{0}}{\partial \Phi }\;e_{r}+\{(\cos^{2}\alpha +\frac{\sin^{2}\alpha }{C})\;\frac{\partial w_{0}}{\partial \Theta }-\frac{\partial V_{0}}{\partial z}\}\;e_{f}-\frac{\partial w_{0}}{\partial \Phi }\;e_{d}.$$

The discrepancy in the directions ${\text{e}}_{d}$ and ${\text{e}}_{r}$ between the expression (5.31) and the vorticity vector (5.30), which is the appropriate one for the underlying fluid flow, is due to the fact that the transformation (4.12) involves a stretching along the ${\text{e}}_{f}$ direction, which does not alter the ${\text{e}}_{f}$ component of the vorticity but intensifies the other two components, changing their leading order. Therefore, from the curl of the velocity of the model equation (5.28) we can recover only the ${\text{e}}_{f}$ component of the vorticity of the original system. Note that one immediate consequence of (5.30) is that the flow is irrotational only if $w_{0}$ does not depend on Θ, and $V_{0}$ does not depend on both Θ and z. Such a restriction makes it impossible for the type of waves that we seek to exist within the irrotational framework. Correspondingly, with regard to (5.28), the constraint

$$\frac{\partial w_{0}}{\partial X}=\frac{\partial V_{0}}{\partial z},\;\text{i.e.}\;(\cos^{2}\alpha +\frac{\sin^{2}\alpha }{C})\;\frac{\partial w_{0}}{\partial \Theta }=\frac{\partial V_{0}}{\partial z},$$

expressing the irrotationality of the $\left(V_{0},\;w_{0}\right)$-flow in the (X,z)-variables, for fixed values of Φ, is also very restrictive. We also note that the inviscid assumption is not adequate for atmospheric flows in the lower troposphere. We conclude, therefore, that any models which build on irrotational inviscid flow (such as the Korteweg–de Vries and Benjamin–Ono equations) cannot be regarded as relevant or reliable descriptions of phenomena such as the morning glory.

With this derivation and these observations in place, we now study the nonlinear wave equation, (5.28), in the light of the many observations of waves similar to the morning glory, and relate its solution to the associated properties of the atmosphere that make these type of phenomena possible.

## 6. Nonlinear wave propagation

The main purpose of this work has been to obtain, using systematic asymptotic methods, an equation for nonlinear wave propagation that captures the essential elements of morning glory-type waves, incorporating all the relevant dynamics and thermodynamics without recourse to additional simplifications. Equation (5.28) has been derived and the obvious next stage is to solve it, aiming to find relevant solutions. This, however, has proved impossible in sufficient generality to produce the detailed structure of waves such as the morning glory; nevertheless, there is much that we can deduce from our formulation of this wave problem, all of which sheds light on its dynamics. In addition, we are able to provide some compelling arguments for indicating that (5.28) is relevant to the description of nonlinear waves in the atmosphere and, as such, is worthy of further investigation (some of which will probably need to be numerical).

### (a) Direction of propagation of the waves

An unexpected prediction from our theory concerns the direction of propagation of the waves described by equation (5.28). It is clear from equation (5.25) that both the material-derivative term and the viscous term vanish for $\alpha \in \{0,\pm \frac{\pi }{2}\}$ and so a nonlinear wave (with any associated dissipation) cannot exist within our asymptotic formulation for these specific angular values. In particular, for $\alpha =\pm \frac{\pi }{2}$, we obtain

$$V_{0}=-\frac{K}{2S}\;\text{and}\;C\;\frac{\partial (\rho_{0}w_{0})}{\partial z}+\frac{\partial (\rho_{0}V_{0})}{\partial \Theta }=0,$$

from (5.25) and (5.24), respectively; thus we have a solution, which, in its most general form, is a uniform flow with

$$V_{0}=-\frac{K(\Phi ,z,t)}{2\sin(\theta_{0}\pm \Phi )},\;\rho_{0}w_{0}=L(\Phi ,\Theta ,t),$$

where L is an arbitrary function. Similarly, for α=0, we obtain

$$2\cos\theta_{0}(w_{0}\cos\theta_{0}-V_{0}\sin\theta_{0})=-T_{0}g\;\frac{\partial G}{\partial \Phi }\;\text{and}\;\frac{\partial (\rho_{0}w_{0})}{\partial z}+\frac{\partial (\rho_{0}V_{0})}{\partial \Theta }=0\;,$$

which has the solution

$$\rho_{0}V_{0}=N(z\cos\theta_{0}-\Theta \sin\theta_{0})+\frac{g}{\sin(2\theta_{0})}\;p_{0}\;\;\frac{\partial G}{\partial \Phi },$$

and correspondingly for $\rho_{0}w_{0}$, where N is an arbitrary function. We conclude, therefore, that any nonlinear waves that are recovered from our asymptotic procedure must necessarily propagate in directions other than North–South or East–West. Consistent with this conclusion, we note that many morning-glory waves, for example, do not propagate in the azimuthal or meridional directions, particularly when they appear over the open ocean; see, for example, the data presented in [6,28]. This result is based on the waves—or lack of them—being generated by pressure or temperature gradients that exist in the atmosphere by virtue of appropriate heat sources (and so consistent with our equations describing the atmosphere). Any external guides, such as the position of land masses or their orography, are not included in our current model of the Earth; this important extension is left for future investigation.

### (b) Breeze-like flows

We can expect that any useful application of our theory should allow the waves to propagate in the presence of a background flow, e.g. in the presence of a breeze in the lower troposphere (as typically occurs for the morning glory). We thus seek a solution of equation (5.28) in the form Ψ=Ψ(z,Φ), which gives

$$-\sigma S\;\frac{\partial \Psi }{\partial z}-\frac{1}{R_{e}}\;\frac{\partial }{\partial z}(m(z)\;\frac{\partial }{\partial z})(\frac{1}{\rho_{0}}\;\frac{\partial \Psi }{\partial z})=K(z,\Phi ),$$

and, using (5.27), this becomes

6.1 $$\rho_{0}\sigma S\;V_{0}+\frac{1}{R_{e}}\;\frac{\partial }{\partial z}(m(z)\;\frac{\partial V_{0}}{\partial z})=-\{\cos^{2}\alpha +\frac{\sin^{2}\alpha }{C}\}K(z,\Phi ).$$

This equation describes a property of the solution that must hold in any regions where the wave profile asymptotes to uniform conditions, i.e. as Θ→±∞. Thus, in this context, we have a breeze described by a horizontal velocity $V_{0}(z,\Phi )$, which we require to be zero on z=0 (the no-slip condition at the Earth’s surface) and on $z=z_{0}$ (and above it), this being the level where the temperature inversion occurs. This breeze then exists only in a rather thin region above the Earth’s surface. From (5.30), we compute the vorticity

$$\gamma_{0}=-\frac{\partial V_{0}}{\partial z}\;e_{f}\;;$$

this shows that non-trivial flows of this type are never irrotational. A simple model profile is

$$V_{0}(z,\Phi )=4V_{m}(\Phi )\;z/z_{0}(1-z/z_{0})\;,\;0\leq z\leq z_{0}\;,$$

which has maximum speed $V_{m}(\Phi )$ at $z=z_{0}/2$; this function can be chosen to limit the lateral extent of the breeze by requiring that $V_{m}(\Phi )=0$ outside $-\Phi_{0}<\Phi <\Phi_{0}$ (for some $\Phi_{0}>0$). We observe in passing, because $R_{e}$ is large, that the specification of K is dominated by $V_{0}$ alone:

$$K(z,\Phi )\approx -\frac{\sigma S}{\cos^{2}\alpha +\sin^{2}\alpha /C}\;\rho_{0}(z)V_{0}(z,\Phi ).$$

We therefore interpret equation (6.1) as determining K for a given $V_{0}$ at one asymptotic direction in Θ and then, at the other, we could have a different form (based on the solution of equation (6.1), given K), although we are likely to require no more than that the same $V_{0}$ exists throughout. Now, consistent with this formulation, and particularly relevant to the appearance of waves in the presence of an inversion layer, we choose $T_{0}(\zeta )$ so that the temperature increases (typically almost linearly) up to the height at which the inversion occurs (i.e. at $z=z_{0}$). Thereafter, the temperature decreases in the familiar linear fashion according to (5.12).

Thus, given the velocity profile of the breeze, $V_{0}(z,\Phi )$, we can find K(z,Φ), which, from (5.26), determines G(z,Φ); we then calculate ∂G/∂z and so obtain the temperature correction, $T_{1}$, from (5.17). This allows us to write down

$$T=T_{0}[gz+\frac{1}{2}\;\sin^{2}(\theta_{0}+\Phi \sin\alpha +\varepsilon \;\Theta \cos\alpha )]+\varepsilon T_{0}^{2}\;\frac{\partial G}{\partial z}+O(\varepsilon^{2})\;,$$

which describes the temperature in the atmosphere, including any temperature inversion, as well as a contribution from the detailed structure of the breeze. We can also obtain expressions for the pressure, $p∼p_{0}+\varepsilon p_{1}$, from (5.10) and (5.15). Conversely, given $T_{0}^{2}\;(\partial G/\partial z)$ as the deviation away from the background-temperature profile, we may, through a sequence of routine integrations, determine G, then K and finally the associated breeze, $V_{0}(z,\Phi )$. In some locations this breeze always exists throughout the region where the nonlinear wave evolves while, in others, equation (6.1) can only be solved for certain types of forcing K.

For example, the geographical coordinates of the Gulf of Carpentaria being 14${\;}^{\circ }$S 139${\;}^{\circ }$E, we have C≈0.97, S≈−0.24 and σ≈133 for α=5π/4 (suitable for a breeze propagating in the south-west direction), so that we can write (6.1) in the form

6.2 $$\beta \;V_{0}-\frac{\partial }{\partial s}(\hat{m}(s)\;\frac{\partial V_{0}}{\partial s})=k_{0}(s,\Phi ),\;0<s<1,$$

with

$$\begin{array}{rl} \beta & \;=-\sigma SR_{e}>0,\;s=\frac{1}{\int_{0}^{z_{0}}\rho_{0}(\xi )\;d\xi }\;\int_{0}^{z}\rho_{0}(\xi )\;d\xi ,\\ \hat{m}(s) & \;=\frac{\rho_{0}(z)m(z)}{(\int_{0}^{z_{0}}\rho_{0}(\xi )\;d\xi {)}^{2}},\;k_{0}(s,\Phi )=R_{e}\{\cos^{2}\alpha +\frac{\sin^{2}\alpha }{C}\}\;\frac{K(z,\Phi )}{\rho_{0}(z)}. \end{array}$$

For every fixed Φ, the boundary conditions associated with (6.2) are $V_{0}=0$ at s=0 and at s=1: this is a regular Sturm–Liouville problem [29]. Assuming that $\rho_{0}$ and K are continuous functions, while m is continuously differentiable, the self-adjoint unbounded linear operator $S=\beta -(\partial /\partial s)(\hat{m}(s)\;\frac{\partial }{\partial s})$, acting in $L^{2}(0,1)$ with domain the Sobolev space $H_{0}^{2}(0,1)=\{V\in H^{2}(0,1):V(0)=V(1)=0\}$, has a discrete spectrum with simple eigenvalues $\lambda_{1}<\lambda_{2}<\ldots <\lambda_{n}<\ldots$ accumulating at infinity, and with the corresponding eigenfunctions ${\left\{f_{n}\right\}}_{n\geq 1}$ forming an orthonormal basis of $L^{2}(0,1)$; see [30]. Multiplying both sides of $Sf_{1}=\lambda_{1}f_{1}$ by $f_{1}$, an integration on (0,1) yields

$$\int_{0}^{1}\hat{m}(s)\;{\left[f_{1}^{\prime }(s)\right]}^{2}\;ds=\lambda_{1}-\beta ,$$

after performing an integration by parts. Therefore $\lambda_{1}>\beta$, so that zero is not an eigenvalue of S. The inverse $S^{-1}$ of S being compact and self-adjoint, the Fredholm alternative now ensures that the equation SV=k has, for every $k\in L^{2}(0,1)$, a unique solution

$$V(s)=\int_{0}^{1}G(s,\tilde{s})\;k(\tilde{s})\;d\tilde{s}=\sum_{n=1}^{\infty }\frac{1}{\lambda_{n}}\;\langle f_{n},\;k\rangle \;f_{n},$$

in $L^{2}(0,1)$, expressed in terms of the Green function

$$G(s,\tilde{s})=\sum_{n=1}^{\infty }\frac{1}{\lambda_{n}}\;f_{n}(s)\;f_{n}(\tilde{s});$$

here ⟨⋅,⋅⟩ is the scalar product of $L^{2}(0,1)$. Moreover, if $\rho_{0}$, m and K are smooth, Sobolev inequalities ensure that this solution is also smooth [30]. This argument has demonstrated that the breeze solution exists in the Gulf of Carpentaria under very general conditions, not restricted by special choices of the flow properties. Note that the high-resolution numerical simulations in [6] indicate that the orography representative of the Cape York Peninsula is not a major factor in the generation of the morning glory.

In contrast to the situation described above, in some cases β<0 in (6.2) might lead to zero being an eigenvalue of the operator S; for example, if $m(z)=m_{0}{\left(\int_{0}^{z_{0}}\rho_{0}(\xi )\;\text{d}\xi \right)}^{2}/\rho_{0}(z)$ for some $m_{0}>0$, then $\hat{m}\equiv m_{0}$ and the eigenvalues are $\lambda_{k}=\beta +m_{0}\pi^{2}k^{2}$, with corresponding eigenfunctions $f_{n}(s)=\sqrt{2}\;\sin(k\pi s)$ for k≥1. If n≥1 is such that $\lambda_{n}=0$, then the Fredholm alternative ensures that equation (6.2) can be solved if and only if $\left\langle k_{0}(\cdot ,\Phi \right)$, $f_{n}\rangle =0$. For example, in the context of the roll cloud over land photographed in Calgary on 18 June 2013 (figure 1*a*), its propagation in the North–West direction corresponds to α=π/4, and since the geographical coordinates of the Calgary are 51${\;}^{\circ }$N 114${\;}^{\circ }$W, we have C≈0.62, S≈0.77 and σ≈8.5, so that $\beta =-\sigma SR_{e}<0$. This example may, at first sight, appear to be unrelated to a sea breeze: Calgary is far from any coastal region. However, sea breeze-like flows can arise in landlocked regions. In this case, cool air, driven by the downdraft associated with a thunderstorm’s cloud, spreads out in an outflow that undercuts the warm air being drawn upwards by the storm’s updraft (see also the discussion in [31,32]). These are precisely the general conditions described earlier as pertaining to offshore and onshore breezes associated with the morning glory. Further, as the cool air lifts the moist, warm air, water condenses and may create a roll wave (as observed on 18 June 2013).

### (c) Bore-like flows

We now discuss a special solution which admits one of the crucial properties that we expect: a rapid vertical displacement of the flow streamline, similar to that of the free surface in river bores [33]. For this, we seek a solution of (5.28) in the form

6.3 Ψ(Θ,z,t;Φ)=A(z,t;Φ)+B(z,t;Φ) η(Θ,t;Φ),

and then we make the choices

6.4 $$\begin{array}{rl} & \frac{\partial^{2}B}{\partial z\partial t}-\sigma S\;\frac{\partial B}{\partial z}-\frac{1}{R_{e}}\;\frac{\partial }{\partial z}\{m(z)\;\frac{\partial }{\partial z}\}(\frac{1}{\rho_{0}}\;\frac{\partial B}{\partial z})=0\;, \end{array}$$

6.5 $$\begin{array}{rl} & \frac{1}{\rho_{0}}\;\frac{\partial A}{\partial z}\frac{\partial B}{\partial z}-B\;\frac{\partial }{\partial z}(\frac{1}{\rho_{0}}\;\frac{\partial A}{\partial z})-\sigma BC\cos\alpha =0, \end{array}$$

6.6 $$\begin{array}{rl} & \frac{1}{\rho_{0}}(\frac{\partial B}{\partial z}{)}^{2}-B\;\frac{\partial }{\partial z}(\frac{1}{\rho_{0}}\;\frac{\partial B}{\partial z})=a(\Phi )\;\frac{\partial B}{\partial z} \end{array}$$

6.7 $$\begin{array}{rl} \;\mathrm{and}\; & \frac{M(z)}{R_{e}}\;\frac{1}{\rho_{0}}\;(\cos^{2}\alpha +\frac{\sin^{2}\alpha }{C})=\nu (\Phi )\;, \end{array}$$

with the condition (6.7) implying that the horizontal kinematic eddy viscosity, $M/\rho_{0}$, varies, at most, as a function of Φ. Further, we set

$$\hat{K}(t;\;\Phi )=\frac{1}{\partial B/\partial z}\;\{K(z,t;\;\Phi )-\frac{\partial^{2}A}{\partial z\partial t}+\sigma S\;\frac{\partial A}{\partial z}+\frac{1}{R_{e}}\;\frac{\partial }{\partial z}(m(z)\;\frac{\partial }{\partial z})(\frac{1}{\rho_{0}}\;\frac{\partial A}{\partial z})\},$$

which restricts the form taken by the forcing function K, so that (5.28) becomes

6.8 $$\frac{\partial \eta }{\partial t}+a\;\eta \;\frac{\partial \eta }{\partial \Theta }=\nu \;\frac{\partial^{2}\eta }{\partial \Theta^{2}}+\hat{K}(t;\;\Phi ).$$

Equation (6.8) is essentially a Burgers equation, which can be recast as such by writing

$$\eta (\Theta ,t;\;\Phi )=\int_{0}^{t}\hat{K}(t^{\prime };\;\Phi )\;dt^{\prime }+X(\xi ,t;\;\Phi ),\;\xi =\Theta +f(t;\;\Phi ),$$

which gives

6.9 $$\frac{\partial X}{\partial t}+aX\;\frac{\partial X}{\partial \xi }=\nu \;\frac{\partial^{2}X}{\partial \xi^{2}},$$

for

$$f(t;\;\Phi )=-a(\Phi )\;\int_{0}^{t}\int_{0}^{t^{\prime }}\hat{K}(t^{\prime \prime };\;\Phi )\;dt^{\prime \prime }dt^{\prime }.$$

The problem is therefore reduced to a classical Burgers equation, but with special choices (as is to be expected); we now examine what these restrictions imply.

From equation (6.6), we find that

6.10 $$B(z,t;\;\Phi )=-\frac{a(\Phi )}{\lambda (t;\;\Phi )}\;+b(t;\;\Phi )\;\exp[\lambda (t;\;\Phi )\int_{0}^{z}\rho_{0}(z^{\prime })\;dz^{\prime }],$$

where b and λ are arbitrary functions.

The degenerate case, λ=0, produces the solution

6.11 $$B(z,t;\;\Phi )=\hat{b}(t;\;\Phi )+a(\Phi )\int_{0}^{z}\rho_{0}(z^{\prime })\;dz,$$

where $\hat{b}$ is arbitrary. Knowing B, we see that equation (6.5) gives

6.12 $$\begin{array}{rl} A(z,t;\;\Phi ) & \;=A_{0}(t;\;\Phi )\int_{0}^{z}\rho_{0}(z^{\prime })B(z^{\prime },t;\;\Phi )\;dz^{\prime }\\ & \;\;-\sigma C\cos\alpha \int_{0}^{z}\int_{0}^{z^{\prime }}\frac{\rho_{0}(z^{\prime })B(z^{\prime },t;\;\Phi )}{B(z^{\prime \prime },t;\;\Phi )}\;dz^{\prime \prime }dz^{\prime }+A_{1}(t;\;\Phi ), \end{array}$$

where $A_{0}$, $A_{1}$ are arbitrary functions. Finally, equation (6.4) determines the vertical eddy viscosity which is consistent with these various choices (and which must be a function of z, but with parametric dependence on Φ, if required); using (6.10), we find that

6.13 $$\frac{m(z)}{R_{e}}=\frac{1}{\rho_{0}}\;\{\frac{\beta -\sigma S}{\lambda^{2}}-\gamma \;\exp[-\lambda \int_{0}^{z}\rho_{0}(z^{\prime })\;dz^{\prime }]\}\;,$$

where γ(Φ) is arbitrary,

$$\beta (\Phi )=\frac{1}{b}\;\frac{db}{dt},$$

and we must have λ=λ(Φ) for m to be independent of t. On the other hand, if we use (6.11), then we see that, although m(z) is arbitrary, we must take a=0, i.e. the nonlinear term is absent from the Burgers equation—and we anticipate that nonlinear evolution is an important ingredient.

For λ≠0 the Burgers equation (6.8) admits bore-like solutions, for example, the travelling wave

$$\eta (\Theta ,t)=\frac{c}{a}-\frac{2\gamma b}{a}\;\tanh[b(\Theta -ct)]\;\text{with}\;bc\neq 0,$$

if $\hat{K}\equiv 0$, and so, from (6.3), we have a corresponding solution of our model equation, (5.28). However, solutions of this form are valid only in a neighbourhood of the thermal inversion layer and have to be matched with suitable air flows in the regions of the troposphere below and above it. Indeed, using (6.10) and (6.12), with the constraints just noted, we see that (5.27) gives

6.14 $$w_{0}=-\frac{1}{\rho_{0}}\;\{-\frac{a}{\lambda }+b_{0}\;e^{\beta t}\;\exp[\lambda \int_{0}^{z}\rho_{0}(z^{\prime })\;dz^{\prime }]\}\;\frac{\partial X}{\partial \xi },$$

where $b_{0}(\Phi )$ is arbitrary. We may impose $w_{0}=0$ on z=0 at all times t, by choosing β=0 and $b_{0}=a/\lambda$. Correspondingly, we obtain

6.15 $$\begin{array}{rl} (\cos^{2}\alpha +\frac{\sin^{2}\alpha }{C})\;V_{0} & \;=-\;\frac{a}{\lambda }(1-\exp[\lambda \int_{0}^{z}\rho_{0}(z^{\prime })\;dz^{\prime }])(A_{0}(t;\;\Phi )-\sigma C\cos\alpha \int_{0}^{z}\frac{dz^{\prime }}{B(z^{\prime },t;\;\Phi )})\\ & \;\;+a\;\exp[\lambda \int_{0}^{z}\rho_{0}(z^{\prime })\;dz^{\prime }](\int_{0}^{t}\hat{K}(t^{\prime },\;\Phi )\;dt^{\prime }+X(\xi ,t;\;\Phi ))\;, \end{array}$$

but we are unable to impose the no-slip condition here (because the necessary z-derivatives have been absorbed into the definition of our special viscosity); indeed, on z=0, we have

$$(\cos^{2}\alpha +\frac{\sin^{2}\alpha }{C})\;V_{0}=a(\int_{0}^{t}\hat{K}(t^{\prime },\;\Phi )\;dt^{\prime }+X(\xi ,t;\;\Phi )).$$

Thus we may have breezes described by the behaviour of X far ahead, and far behind, the wavefront (as mentioned earlier), but their vertical structure is prescribed in this special, exact solution. From (6.13), a requirement for this special solution to exist is that

6.16 $$\frac{m(z)}{R_{e}}=-\frac{1}{\rho_{0}}\;\{\frac{\sigma S}{\lambda^{2}}+\gamma \;\exp[-\lambda \int_{0}^{z}\rho_{0}(z^{\prime })\;dz^{\prime }]\},$$

where we may choose γ and λ to produce a reasonable model for the variation of the vertical dynamic eddy viscosity in the lowermost region of the troposphere. Finally, we may compute the vorticity associated with this bore-like flow. At leading order, we find that

$$\begin{array}{rl} \gamma_{0} & \;=(-(C\cos^{2}\alpha +\sin^{2}\alpha )\;\frac{B}{C\rho_{0}}\;\frac{\partial^{2}\eta }{\partial \Theta^{2}}+\frac{C[\partial /\partial z((1/\rho_{0})(\partial A/\partial z))+\eta \;(\partial /\partial z)((1/\rho_{0})(\partial B/\partial z))\;]}{C\cos^{2}\alpha +\sin^{2}\alpha })\;e_{f}\\ & \;\;+(C-1)\sin\alpha \cos\alpha \;\frac{B}{C\rho_{0}}\;\frac{\partial^{2}\eta }{\partial \Theta^{2}}\;e_{d}+\frac{(C-1)\sin\alpha \cos\alpha }{(C\cos^{2}\alpha +\sin^{2}\alpha )\rho_{0}}\;\frac{\partial B}{\partial z}\;\frac{\partial \eta }{\partial \Theta }\;e_{r}. \end{array}$$

This shows that the flow cannot, in any circumstances, be regarded as irrotational; indeed, even with the special choice α=0 (which can never correspond to a morning glory wave) there is a component of vorticity perpendicular to the direction of travel.

### (d) Oscillatory-like solution

In §2, we described the general properties of the morning glory clouds, which are often seen as a sequence of such clouds; to generate these, an oscillatory structure is required. This type of flow will then provide regions of updraft and downdraft that allow the creation of a wave train of clouds. We now show that our model equation (5.28) possesses solutions of the appropriate form, although to extract this property as an exact solution requires a few simplifying assumptions. We expect that such a solution will be relevant to a neighbourhood of the thermal inversion layer, about which there will be only small changes in the density and viscosities of the air. Indeed, the field data in [1] show that the density variation across the thermal inversion layer is only about 1% and so we can take advantage of this, if expedient. In this discussion of equation (5.28), we first allow the background density, $\rho_{0}(gz+\frac{1}{2}\;S^{2})$, to take any (reasonable) general form, and then introduce

6.17 $$Z=\int_{0}^{z}\rho_{0}(gz^{\prime }+\frac{1}{2}\;S^{2})\;dz^{\prime }.$$

We now transform equation (5.28), treating Ψ=Ψ(Φ,Θ,Z,t), to give

6.18 $$\begin{array}{rl} & \frac{\partial^{2}\Psi }{\partial Z\partial t}+\frac{\partial \Psi }{\partial Z}\;\frac{\partial^{2}\Psi }{\partial Z\partial \Theta }-\frac{\partial \Psi }{\partial \Theta }\;\frac{\partial^{2}\Psi }{\partial Z^{2}}-\sigma (S\;\frac{\partial \Psi }{\partial Z}+\frac{C\cos\alpha }{d_{0}}\;\frac{\partial \Psi }{\partial \Theta })\\ & \;\;-\frac{1}{R_{e}}\;\{\frac{\partial }{\partial Z}\;(\hat{m}\;\frac{\partial }{\partial Z})+\hat{M}\;(\cos^{2}\alpha +\frac{\sin^{2}\alpha }{C})\frac{\partial^{2}}{\partial \Theta^{2}}\}\frac{\partial \Psi }{\partial Z}=\hat{K}(\Phi ,Z,t), \end{array}$$

where

6.19 $$\rho_{0}=d_{0}(\Phi ,Z),\;m\rho_{0}=\hat{m}(\Phi ,Z),\;\frac{M}{\rho_{0}}=\hat{M}(\Phi ,Z)\;,\;\frac{K}{\rho_{0}}=\hat{K}(\Phi ,Z,t).$$

We note that the positivity of the density function allows us to redefine this function in terms of Z; the other definitions are required for consistency (and are therefore part of the process of simplification needed to develop this special solution). To proceed, we seek a solution

6.20 $$\Psi (\Phi ,\Theta ,Z,t)=\tau (\Phi ,Z)+\chi \;e^{kZ}\cos[k(\Theta -s_{0}\;t)],$$

where χ, k and $s_{0}$ depend on only Φ. (The addition of a term proportional to Θ is possible, but this generates a uniform flow in the vertical direction which is not appropriate for the phenomena that we are examining here.) We find that (6.20) solves (6.18) if

6.21 $$\begin{array}{rl} & \frac{\partial^{2}\tau }{\partial Z^{2}}-k\;\frac{\partial \tau }{\partial Z}=-ks_{0}-\frac{\sigma C\cos\alpha }{d_{0}}, \end{array}$$

6.22 $$\begin{array}{rl} & k^{2}\;\hat{M}(\cos^{2}\alpha +\frac{\sin^{2}\alpha }{C})-k(\frac{\partial \hat{m}}{\partial Z}+k\;\hat{m})=\sigma SR_{e} \end{array}$$

6.23 $$\begin{array}{rl} \;\mathrm{and}\; & \hat{K}=-\sigma S\;\frac{\partial \tau }{\partial Z}-\frac{1}{R_{e}}\;\frac{\partial }{\partial Z}(\hat{m}\;\frac{\partial^{2}\tau }{\partial Z^{2}}). \end{array}$$

Now, for equation (6.22) to be consistent, we set $\hat{M}=\hat{M}(\Phi )$ and $\hat{m}=\hat{m}(\Phi )$ (although a slightly more general version is allowed with $\partial \hat{m}/\partial Z+k\;\hat{m}$ a function of only Φ), to give

$$k^{2}=\frac{\sigma SR_{e}}{\hat{M}(\cos^{2}\alpha +\frac{\sin^{2}\alpha }{C})-\hat{m}}\;,$$

and oscillatory solutions require

$$\hat{M}(\cos^{2}\alpha +\frac{\sin^{2}\alpha }{C})>\hat{m}.$$

Thus, we have shown that a solution of the form (6.20) exists, where

$$\tau =s_{0}Z-\sigma C\cos\alpha \int_{0}^{Z}\int_{0}^{Z^{\prime }}\frac{e^{k(Z^{\prime }-Z^{\prime \prime })}}{d_{0}(\Phi ,Z^{\prime \prime })}\;dZ^{\prime \prime }dZ^{\prime }+A\;e^{kZ}+B,$$

is determined from equation (6.21), given the density function $d_{0}(\Phi ,Z)$ and a suitable choice of wave speed, $s_{0}$; here A(Φ) and B(Φ) are arbitrary. This, in turn, enables the forcing function, $\hat{K}(\Phi ,Z)$, to be found from equation (6.23), and then $\hat{K}$ can be used to identify the heat sources for this flow. However, this solution does not exist if the terms that do not follow the Burgers pattern in (5.28) are absent, i.e. for σ=0 we have k=0. This property of our special solution indicates that the terms involving σ play a critical rôle in confirming the existence of an oscillatory component to the available solutions. Further, the solution that we describe here is nonlinear, in the sense that the horizontal background flow

$$\frac{\partial \tau /\partial Z}{d_{0}(\cos^{2}\alpha +\sin^{2}\alpha /C)},$$

depends on both the wavenumber and the wave speed (via τ), although it is independent of the wave amplitude, χ. Indeed, this solution also allows for the specification of the wave speed independently of the wavenumber determined above. The vertical velocity component

$$w_{0}=\frac{k\chi }{d_{0}}\;e^{kZ}\sin[k(\Theta -s_{0}\;t)],$$

shows that we do have alternating regions of updraft and downdraft, as time passes, heralding the formation of the sequence of roll waves. Furthermore, associated with this oscillatory solution, we have the vorticity

$$\begin{array}{rl} \gamma_{0} & \;=\{(C\cos^{2}\alpha +\sin^{2}\alpha )\;\frac{k^{2}\chi }{C\rho_{0}}\;e^{kZ}\;\cos[k(\Theta -s_{o}t)]\\ & \;\;-\frac{C\rho_{0}}{C\cos^{2}\alpha +\sin^{\alpha }}(\frac{\partial^{2}\tau }{\partial Z^{2}}+k^{2}\chi \;e^{kZ}\;\cos[k(\Theta -s_{o}t)])\}\;e_{f}\\ & \;\;+\{(1-C)\;\frac{k^{2}\chi }{C\rho_{0}}\;e^{kZ}\;\cos[k(\Theta -s_{o}t)]\;\sin\alpha \cos\alpha \}\;e_{d}\\ & \;\;+\{\frac{(1-C)k^{2}\chi \sin\alpha \cos\alpha }{C\cos^{2}\alpha +\sin^{\alpha }}\;e^{kZ}\;\sin[k(\Theta -s_{o}t)]\}\;e_{r}, \end{array}$$

which again demonstrates that irrotationality is very wide of the mark. Indeed, we see that the horizontal background flow (given by τ) has vorticity aligned only with the wavefront, whereas the oscillatory component generates a far more complicated flow, with vorticity components in all three directions.

## 7. Discussion

The investigation that we have described here provides a derivation of a nonlinear wave equation, (5.28), from the full set of governing equations for the atmosphere on Earth, written in rotating, spherical coordinates. So, starting from the equations for a viscous, compressible fluid, coupled to a suitable prescription of the thermodynamics of the atmosphere (an equation of state and the first law), the equations for unsteady motion (using the timescale $1/\Omega^{\prime }\approx 3\frac{1}{2}$ h) have been non-dimensionalized and the thin-shell parameter (ε) introduced; all other physical properties are represented by suitable parameters, which are held fixed as ε→0. To consider atmospheric waves propagating in a particular direction over the surface of a spherical Earth, we prescribe scales associated with the direction of propagation, and with the extent of the wavefront. In terms of the fundamental parameter used for the construction of the asymptotic expansion (ε), a natural choice is to make the scale in the propagation direction (defined by Θ) O(ε) smaller than that along the wavefront (defined by Φ). Not only is this reasonable on physical grounds, but it is almost essential as we aim to derive a nonlinear wave equation consistent with the higher-order perturbation terms. This introduction of a single parameter, and the scaling associated with it, is the sole basis for the development of the asymptotic solution, i.e. no other simplifying assumptions are invoked. The general pattern for the asymptotic development then follows the familiar route when perturbing a leading-order solution.

Here, however, apart from the general complexity of the governing equations, the main complication in this type of problem arises from the need to perturb a background state that represents a realistic model of the steady atmosphere. Of course, showing how this is accomplished, using only the thin-shell parameter, is an important part of the development presented here. The leading order produces a description for the background state, which is necessarily steady (on our chosen timescale) and governed, in the main, by its dependence on the vertical coordinate; a solution of this system is the familiar linear reduction of the temperature with height. At the next order, i.e. terms O(ε), we obtain a correction to the background state that determines its Φ-dependence and, in consequence, also the Φ-dependence of the leading-order velocity field. At these first two orders, the leading-order velocity field appears (in the first law) but cannot be determined; in addition, at O(ε), the vertical velocity component $w_{1}$ also appears. Finally, at $\text{O}(\varepsilon^{2})$, we obtain two equations that define the leading-order velocity field $\left(U_{0},V_{0},w_{0}\right)$, given the dominant description of the background state of the atmosphere. That we have all three components appearing here is consistent when we specify that the wave is moving in the Θ-direction only; thus we set $U_{0}\equiv 0$ and we can then solve for the two-dimensional velocity field $\left(V_{0},w_{0}\right)$, but which can still admit a structure in the Φ-direction. This is best accomplished by introducing a stream function, Ψ, and then obtaining the model equation (5.28), which describes how this evolves. This resembles a generalization of the boundary-layer equations [34,35] and appears to be Burgers-like, although of one higher order, with variable coefficients, with terms not normally associated with a Burgers equation and with a general forcing term (independent of Θ). It is certainly unrealistic to expect that we can find a suitable exact solution which relates directly to observed atmospheric waves. Nevertheless, we have been able to obtain some useful pointers as to the properties and relevance of this equation, although far more investigation is still needed (both analytical and numerical). Furthermore, an important element in the development was to find the leading-order approximation to the vorticity, which provides some fundamental information about the nature of the flows.

Firstly, and quite unexpectedly, the particular asymptotic route that we have followed—others are possible but, we suggest, ours is arguably the most natural and direct—leads to a restriction on the direction of propagation around the sphere. No wave evolution, governed by equation (5.28), is possible in the East–West or the North–South directions. The equation, in these two cases, simply predicts the existence of uniform flows without a wave-like structure. It is possible that wave propagation, of the general type described here, still exists (in our sense of a perturbation of a background state) but where this corresponds to equations which appear at even higher order. We have not investigated this possibility, which, in any event, will certainly involve considerable algebraic complexity.

Secondly, and not at all surprisingly, we have shown that there are special solutions that are independent of Θ and t (and so cannot exhibit wave-like properties). These solutions represent breezes and can take many different forms, being generated by any suitable forcing function, K(z,Φ). Such flows are expected to be relevant both as the initial state before waves are generated, and to give the behaviour some distance away from any oscillatory or bore-like regime. Note that there are several aspects of the dynamics of sea-breezes that remain to be elucidated (see the discussion in [36]).

Thirdly, by seeking a special similarity solution-structure (of the type pursued in [34,37] but with an acceptable physical basis), we have seen that equation (5.28) can be reduced to a standard Burgers equation that admits bore-like solutions. Although this exact solution does require, in addition, some special choices of the variable coefficients (in particular the viscosities) and a relaxation of the bottom boundary condition, the result is encouraging. The fact that the equation can be used to recover bore-like solutions is an important element in applying it to some of the wave phenomena that are observed. While theories for the dynamics of atmospheric bores have largely relied on analogies with those of shallow-water flow (for which we refer to the discussions in [33,38]), the atmospheric case is considerably more complex due to thermodynamical effects associated with density and pressure variations (see [2,39–41] for numerical simulations and field data indicating that the analogy with shallow-water dynamics is quite limited). Indeed, our evaluation of the vorticity shows that this is zero only in the absence of oscillations, and so any model that is based on the assumption of irrotationality must be suspect. Note also that morning-glory clouds typically occur within the atmospheric boundary layer, where viscosity plays a role in the air-flow dynamics.

Finally, because the wave propagation that we are trying to model often appears as a sequence of undulations (which are essential to the creation of the morning-glory cloud patterns), we would want the equation to exhibit some oscillatory properties. We have shown, under a transformation admitting a general background density, that an oscillatory solution does exist for some special choices of the viscosities. This, in the context of a typical morning-glory wave, is the appropriate description of the wave structure that appears around the level of the thermal inversion. Furthermore, this oscillatory solution is absent if the non-Burgers-like terms in equation (5.28), i.e. those terms associated with σ, are removed by setting σ=0. These terms therefore provide a dispersion-like element to the solutions, producing oscillations that are necessary for the existence of solutions that mirror the morning glory as typically observed.

In summary, therefore, the model equation (5.28) appears to possess all the properties that we need: uniform flow, bore-like solutions and oscillatory solutions. However, to combine all these in one solution is beyond us at this stage. This does, however, open the door to a more extensive investigation. So, for example, the construction of numerical solutions might be the way forward; also it might be possible to prove some general results indicating the existence of solutions of the appropriate form satisfying suitable initial and boundary data.

This development also provides us, via the first law of thermodynamics, with the opportunity to identify the heat sources that drive wave systems such as the morning glory. Equation (5.11), the leading-order approximation to the first law, is not particularly informative although, if the temperature does not decrease linearly according to equation (5.12), then there is necessarily a heat source associated with points that move vertically in the atmosphere: a latent-heat contribution. On the other hand, equation (5.18) shows that, in general, we have a far more complicated set of heat sources. Nevertheless, if the background temperature satisfies (5.11), then we have both a moving heat source (via $w_{0}$) and some background (solar) heating. These heat sources are driven by G(Φ,z,t), which can be determined (see (5.26)) if we know K(Φ,z,t), which itself drives the wave motion. Conversely, given the flow structure (in the morning glory, for example), we can determine K, and hence G, and then we may identify the associated heat sources that drive and maintain the motion. An important element in our approach, which has emphasized the dynamical properties of the flow, is that the details of the thermodynamic forcing are not critical to the development. Thus, the nature of the heat sources, and any evaporation or condensation associated with cloud formation, can be added later, as part of the interpretation of the forcing that drives the motion (and makes some of it visible to the observer). This, we suggest, is the most useful way to tackle these problems: produce the best approximation (in the asymptotic sense) to the dynamics, and then identify the detailed thermodynamics and air properties to match the motion.

In conclusion, we have developed, using a systematic asymptotic procedure, a nonlinear wave equation that is consistent with a set of general, governing equations for a compressible fluid. This equation appears, on the basis of the results obtained so far, to possess all the essential properties needed for a description of wave formations such as the morning glory. Of course, more work must be done to elucidate the possible wave structures that can be generated by our nonlinear wave equation. The details presented here, particularly connecting the wave motion to the heat sources, allows for a comprehensive investigation of wave phenomena in the atmosphere. And we have not touched on the role of topography in controlling the wave development: there are, we submit, many avenues that are still to be explored.

## Data Availability

All data used in the paper were published elsewhere.
